# Single-Dose Intranasal Administration of AdCOVID Elicits Systemic and Mucosal Immunity against SARS-CoV-2 and Fully Protects Mice from Lethal Challenge

**DOI:** 10.3390/vaccines9080881

**Published:** 2021-08-09

**Authors:** R. Glenn King, Aaron Silva-Sanchez, Jessica N. Peel, Davide Botta, Alexandria M. Dickson, Amelia K. Pinto, Selene Meza-Perez, S. Rameeza Allie, Michael D. Schultz, Mingyong Liu, John E. Bradley, Shihong Qiu, Guang Yang, Fen Zhou, Esther Zumaquero, Thomas S. Simpler, Betty Mousseau, John T. Killian, Brittany Dean, Qiao Shang, Jennifer L. Tipper, Christopher A. Risley, Kevin S. Harrod, Tsungwei Feng, Young Lee, Bethlehem Shiberu, Vyjayanthi Krishnan, Isabelle Peguillet, Jianfeng Zhang, Todd J. Green, Troy D. Randall, John J. Suschak, Bertrand Georges, James D. Brien, Frances E. Lund, M. Scot Roberts

**Affiliations:** 1 Department of Microbiology, University of Alabama at Birmingham, Birmingham, AL 35294, USA; rgking@uab.edu (R.G.K.); jnpeel@uab.edu (J.N.P.); dbotta@uab.edu (D.B.); mschul20@uab.edu (M.D.S.); qiu@uab.edu (S.Q.); gyang@uab.edu (G.Y.); fzhou@uab.edu (F.Z.); ezm@uab.edu (E.Z.); ssimpler@uab.edu (T.S.S.); mousseau@uab.edu (B.M.); jtkillian@uabmc.edu (J.T.K.J.); bdean819@uab.edu (B.D.); qiaoshang@uabmc.edu (Q.S.); crisley@uab.edu (C.A.R.); tgreen@uab.edu (T.J.G.); 2 Department of Medicine, Division of Clinical Immunology and Rheumatology, University of Alabama at Birmingham, Birmingham, AL 35294, USA; aaronsilvasanchez@uabmc.edu (A.S.-S.); selenemezaperez@uabmc.edu (S.M.-P.); sallie@pennstatehealth.psu.edu (S.R.A.); miliu@uab.edu (M.L.); johnbradley@uabmc.edu (J.E.B.); troyrandall@uabmc.edu (T.D.R.); 3 Department of Molecular Microbiology & Immunology, Saint Louis University, St. Louis, MO 63104, USA; alexandria.dickson@slu.edu (A.M.D.); akpintolab@gmail.com (A.K.P.); brienlab@gmail.com (J.D.B.); 4 Department of Anesthesiology and Perioperative Medicine, University of Alabama at Birmingham, Birmingham, AL 35294, USA; jennifertipper@uabmc.edu (J.L.T.); kevinharrod@uabmc.edu (K.S.H.); 5 Altimmune Inc., Gaithersburg, MD 20878, USA; rfeng@altimmune.com (T.F.); ylee@altimmune.com (Y.L.); bshiberu@altimmune.com (B.S.); vkrishnan@altimmune.com (V.K.); ipeguillet@altimmune.com (I.P.); zhang@altimmune.com (J.Z.); jsuschak@altimmune.com (J.J.S.); bgeorges@altimmune.com (B.G.)

**Keywords:** COVID-19, SARS-CoV-2, receptor binding domain, vaccine, adenovirus vector, viral vector, intranasal, mucosal immunity, IgA

## Abstract

The coronavirus disease 2019 (COVID-19) pandemic has highlighted the urgent need for effective prophylactic vaccination to prevent the spread of severe acute respiratory syndrome coronavirus 2 (SARS-CoV-2). Intranasal vaccination is an attractive strategy to prevent COVID-19 as the nasal mucosa represents the first-line barrier to SARS-CoV-2 entry. The current intramuscular vaccines elicit systemic immunity but not necessarily high-level mucosal immunity. Here, we tested a single intranasal dose of our candidate adenovirus type 5-vectored vaccine encoding the receptor-binding domain (RBD) of the SARS-CoV-2 spike protein (AdCOVID) in inbred, outbred, and transgenic mice. A single intranasal vaccination with AdCOVID elicited a strong and focused immune response against RBD through the induction of mucosal IgA in the respiratory tract, serum neutralizing antibodies, and CD4^+^ and CD8^+^ T cells with a T_h_1-like cytokine expression profile. A single AdCOVID dose resulted in immunity that was sustained for over six months. Moreover, a single intranasal dose completely protected K18-hACE2 mice from lethal SARS-CoV-2 challenge, preventing weight loss and mortality. These data show that AdCOVID promotes concomitant systemic and mucosal immunity and represents a promising vaccine candidate.

## 1. Introduction

First reported in late 2019 in China, severe acute respiratory syndrome coronavirus 2 (SARS-CoV-2) evolved into a global pandemic within a few months [1]. As of this report, the World Health Organization estimates over 178 million cases of coronavirus disease 2019 (COVID-19) worldwide, with over 3.8 million associated deaths [2,3]. Morbidity from SARS-CoV-2 infection can be severe, especially in high-risk groups (e.g., the elderly, individuals with chronic comorbidities such as hypertension, obesity, and diabetes) [4]. Evidence from convalescent COVID-19 patients and survivors of similar β-coronaviruses such as SARS-CoV and Middle East respiratory syndrome (MERS), suggests that COVID-19 survivors may suffer long-term sequelae (e.g., inflammation resulting in damage to the lungs and heart) [5,6,7,8,9]. The global impact on human health and well-being underscores the immediate need for safe and effective vaccines against SARS-CoV-2 to end this pandemic and prevent its return.

Despite the well-recognized role of mucosal immunity in prevention of disease [10,11], all of the COVID-19 vaccines granted emergency use authorization or have been advanced to Phase 3 clinical trials are administered via intramuscular injection [12,13,14,15]. Intramuscular injection elicits systemic immunity but does not result in potent mucosal immune responses. Suboptimal mucosal immunity may limit the utility of intramuscularly administered COVID-19 vaccines since transmission of SARS-CoV-2 is primarily via respiratory droplets released by infected individuals in enclosed spaces [16], with the nose and other portions of the respiratory mucosa being the primary routes of entry [17]. The nasal compartment shows particular susceptibility to SARS-CoV-2 infection due to abundant co-expression of the viral entry receptor (angiotensin-converting enzyme-2, ACE-2) and a required activating protease (TMPRSS2) in nasal goblet and ciliated cells [18]. These cells are thought to be the primary route of infection and it is hypothesized that the nasal cavity serves as the initial reservoir for subsequent seeding of the virus to the lungs [19]. The well-documented association of anosmia with COVID-19 further supports the nasal cavity as a principal reservoir of infection [20], and the presence of high viral load in the nasal cavity may facilitate transmission of the virus, and the emergence of viral variants of concern.

A vaccination route targeting the mucosa presents an attractive alternative to intramuscular delivery. Intranasal delivery may be more appealing to patients as intranasal administration is non-invasive and obviates the need for needles [21]. In addition, data suggest that intranasal delivery may increase vaccine uptake [22], and in contrast to intramuscular injection, mucosal vaccination via the intranasal route has the potential to confer sterilizing immunity in the respiratory tract thereby reducing virus-induced disease and transmission of COVID-19 [23].

Entry of SARS-CoV-2 into host cells depends on engagement of the receptor-binding domain (RBD) of the spike protein to ACE-2, leading to fusion of the virus with the cell membrane [24]. In human convalescent serum, the majority of neutralizing antibodies are directed against the RBD [25,26,27]. While most clinically advanced SARS-CoV-2 vaccine candidates deliver the trimeric spike ectodomain as the target antigen [28,29,30], subdomains of the spike protein such as RBD represent alternative vaccine antigens for stimulation of a more focused immune response targeting well-conserved domains. Such an approach may also limit the induction of non-protective antibodies, mitigating the risk of vaccine-associated enhanced disease (VAED) [31].

Here, we report results of preclinical immunogenicity testing of AdCOVID, an intranasal replication-deficient adenovirus serotype 5 (Ad5)-vectored vaccine candidate against COVID-19 that encodes the RBD from SARS-CoV-2 spike protein. We demonstrate immunogenicity of AdCOVID following a single administration of the vaccine in two strains of mice, which resulted in induction of spike-specific IgG and IgA antibody in sera and bronchoalveolar lavage (BAL) fluids that endured for at least six months. We show functionality of these vaccine-elicited antibodies in live virus neutralization assays. In addition to the induction of robust neutralizing antibody responses and mucosal IgA against SARS-CoV-2, the RBD vaccine candidate stimulated systemic and mucosal cell-mediated immune responses characterized by the induction of type 1 cytokine-producing CD4^+^ and CD8^+^ T cells, including lung-resident memory T cells. Finally, a single administration of AdCOVID completely protected K18-hACE2 mice from lethal challenge. These data support the clinical development of AdCOVID in response to a serious global health threat.

## 2. Materials and Methods

### 2.1. Ethics Statement and Mice

Mice used in these studies were obtained from the Jackson Laboratory (Bar Harbor, ME, USA) (C56BL/6J) or Charles River Laboratories (Las Vegas, NV, USA) (CD-1). K18-hACE2 breeding pairs were originally purchased from Jackson Laboratory (Bar Harbor, ME, USA) and subsequent generations were bred at Saint Louis University (SLU).

### 2.2. Vaccine Candidate

The vaccine candidate evaluated in this study was based on a replication-deficient, E1- and E3-deleted adenovirus type 5 vector platform [32] and expresses a human codon-optimized gene for the RBD domain (residues 302 to 543) of SARS-CoV-2 spike protein (accession number QHD43416.1). The Ad5-vectored RBD transgene includes a human tissue plasminogen activator leader sequence and is expressed under the control of the cytomegalovirus immediate early promoter/enhancer. An initial seed stock was obtained from transfection of recombinant vector plasmid into E1-complementing PER.C6 cells using a scalable transfection system (MaxCyte STX-100; MaxCyte, Gaithersburg, MD, USA) [33]. Following vector expansion, replication-deficient vector was obtained from infected cell lysates and was purified over a CsCl gradient, dialyzed against a formulation buffer containing 10 mm Tris (pH 7.4), 75 mM NaCl, 1 mM MgCl_2_, 10 mM histidine, 5% (wt/vol) sucrose, 0.02% polysorbate-80 (wt/vol), 0.1 mM EDTA, and 0.5% (vol/vol) ethanol and then frozen and stored at −65 °C.

### 2.3. Adenovirus Vaccine Titer Measurement

293 HEK cells were seeded in a 96-well plate one day before Ad vector infection. After inoculation of the appropriate dilutions of adenovirus control and test sample (s) onto duplicate wells, the infected cells were incubated for 3 days. At the end of the infection period, media were removed, and cells were fixed with cold methanol. Following drying and rinsing with PBS, mouse anti-adenovirus-5 hexon antibody was added to each well of cells and incubated at 37 °C for at least 60 min. After removal of the mouse anti-adenovirus-5 hexon antibody and additional PBS washes, HRP-conjugated rat anti-mouse antibody was added to each well and incubated at least 60 min at 37 °C. After removal of the detection antibody and additional PBS washes, cells were stained with DAB (3,3-diaminobenzidine) working solution for at least 10 min. After removal of DAB working solution and additional washing steps with PBS, the stained foci were enumerated using a microscope with a 20× objective.

### 2.4. Vaccination

Female C57BL/6J and CD-1 mice of at least 6 weeks of age were randomly allocated into vaccination groups for immunogenicity experiments. Male and female K18-hACE2 mice of at least 6 weeks of age were equally allocated into vaccination groups for the SARS-CoV-2 challenge experiment. Replication-deficient Ad5 vector encoding the RBD (AdCOVID) from the SARS-CoV-2 spike protein was administered intranasally at the described doses in a volume of 50 µL (25 µL per nostril). Briefly, mice were sedated with isoflurane and placed in the supine position. AdCOVID was delivered into the nasal mucosa by pipette during normal inhalation, and mice were allowed to recover naturally. The control group received 50 µL of vehicle alone by intranasal administration.

### 2.5. Serum Collection

Blood samples were collected from the submandibular vein of vaccinated mice into BD Microtainer blood collection tubes (BD Biosciences, East Rutherford, NJ, USA). The samples were centrifuged at 13,000 rpm at RT for 8–10 min and the serum was collected, aliquoted and frozen at −80 °C until analyzed.

### 2.6. Tissue Processing and Single Cell Isolation

Spleen, medLN, and lung tissues were isolated from vaccinated mice at the indicated timepoints. Lung tissue was minced and then digested in RPMI-1640 medium containing collagenase (1.25 mg/mL; Millipore-Sigma, Burlington, MA, USA) and DNase I (150 units/mL; Millipore-Sigma) for 30 min at 37 °C. To generate single-cell suspensions, digested lung, spleen, and draining lymph nodes were passed through a fine wire mesh. The single-cell suspensions were treated with red blood cell lysis buffer and filtered (100 µm) to remove debris.

### 2.7. BAL Collection

BAL samples were collected using ethyl vinyl acetate (EVA) microbore tubing (Cole-Parmer, Vernon Hills, IL, USA) with inner and outer diameters of 0.02 in and 0.06 inches, respectively. One end of the tube was fitted to a 23G syringe needle attached to a 3-way stopcock. The other end of the tube was inserted postmortem into an incised trachea. BAL fluid was collected as a single 1 mL lavage fraction using ice-cold phenol-free Hank’s Buffered Salt Solution (HBSS, without Ca^2+^ and Mg^2+^) containing 2 mM EDTA. BAL cells were separated from the supernatant by centrifugation at 1800 rpm for 5 min at 4 °C.

### 2.8. Recombinant SARS-CoV-2 Protein Production

To produce recombinant SARS-CoV-2 spike ectodomain protein, two human codon-optimized constructs were generated with linear sequence order encoding: a human IgG leader sequence, the SARS-CoV-2 spike ectodomain (amino acids 14-1211), a GGSG linker, T4 fibritin foldon sequence, a GS linker, and finally an AviTag (construct 1) or 6X-HisTag (construct 2). Each construct was engineered with two sets of mutations to stabilize the protein in a pre-fusion conformation. These included substitution of RRAR > SGAG (residues 682 to 685) [34] at the S1/S2 cleavage site and the introduction of two proline residues; K983P, V984P [34,35]. Avi/His-tagged trimers were produced by co-transfecting plasmid constructs 1 and 2 (1:2 ratio) into FreeStyle 293-F cells. Cells were grown for three days, and the supernatant (media) was recovered by centrifugation. Recombinant spike trimers were purified from media by Fast protein liquid chromatography (FPLC) using a HisTrap HP Column (GE Biosciences, Niskayuna, NY, USA) and elution with 250 mM of imidazole. After exchanging into either 10 mM Tris-HCl, pH 8.0 or 50 mM Bicine, pH 8.3, purified spike ectodomain trimers were biotinylated by addition of biotin-protein ligase (Avidity, La Jolla, CA, USA). Biotinylated spike ectodomain trimers were buffer exchanged into PBS, sterile filtered, aliquoted, then stored at −80 °C until used.

### 2.9. SARS-CoV-2 Spike Cytometric Bead Array

To generate the spike cytometric bead array (CBA), recombinant SARS-CoV-2 ectodomain trimers were passively absorbed onto streptavidin functionalized fluorescent microparticles (3.6 µm; Spherotech, Lake Forest, IL, USA). 500 µg of biotinylated SARS2-CoV-2 was incubated with 2 × 10^7^ treptavidin functionalized fluorescent microparticles in 400 µL of 1% BSA in PBS. Following coupling, the SARS-CoV-2 spike conjugated beads were washed twice in 1 mL of 1% BSA, PBS, 0.05% NaN3, resuspended at 1 × 10^8^ beads/mL and stored at 4 °C. The loading of recombinant SARS2-CoV-2 spike onto the beads was evaluated by staining 1 × 10^5^ beads with dilutions ranging from 1 µg/mL to 2 ng/mL of the recombinant anti-SARS spike antibody CR3022 and visualized with an anti-human IgG secondary antibody.

### 2.10. CBA IgG and IgA Standards

IgG and IgA standards were generated by covalent coupling of isotype-specific polyclonal antibodies to fluorescent particles. Briefly, 0.2 mg of goat polyclonal anti-mouse IgG (SouthernBiotech, Birmingham, AL, USA), anti-IgM (SouthernBiotech, Birmingham, AL, USA), and anti-IgA (SouthernBiotech, Birmingham, AL, USA) antibodies in PBS were mixed with 5 × 10^7^ fluorescent microparticles each with a unique fluorescent intensity in the far-red channels (3.6 µm; Spherotech) resuspended in 0.1 M MES buffer pH 5.0. An equal volume of EDC (1-Ethyl-3-(3-dimethylaminopropyl)-carbodiimide), 10 mg/mL, in 0.1 M MES (2-(N-morpholino) ethanesulfonic acid) buffer pH 5.0 was added and the mixture was incubated overnight at RT. The beads were washed twice by pelleting by centrifugation and resuspension in PBS. Following washing, beads were resuspended in 1% BSA in PBS with 0.005% NaN_3_ as a preservative.

### 2.11. CBA Measurement of Spike-Specific IgG and IgA Responses

The quantification of SARS-CoV-2 spike IgG and IgA was performed in serum or BAL samples obtained from immunized animals using the spike CBA described above. BAL samples (diluted 1/4–8) or serum samples (diluted to 1/1000–5000) in 50 µL of PBS were arrayed in 96-well u-bottom polystyrene plates along with 50 µL of standards consisting of either mouse IgG, IgM, or IgA ranging from 1 µg/mL to 2 ng/mL at 0.75× dilutions (SouthernBiotech, Birmingham, AL, USA). 5 µL of a suspension containing 5 × 10^5^ of each SARS-CoV-2 spike, anti-IgM, anti-IgA, and anti-IgG beads was added to the diluted samples. The suspensions were mixed by pipetting and incubated for 15 min at RT. The beads were washed by the addition of 200 µL of PBS and centrifuged at 3000× *g* for 5 min at RT. The CBA particles were resuspended in a secondary staining solution consisting of polyclonal anti-IgG 488 (SouthernBiotech, Birmingham, AL, USA), and either a goat polyclonal anti-IgM (SouthernBiotech, Birmingham, AL, USA) or anti-IgA (SouthernBiotech, Birmingham, AL, USA) conjugated to PE diluted 1/400 in 1% BSA in PBS. The suspension was incubated for 15 min in the dark at RT. The beads were washed by the addition of 200 µL of PBS and pelleted by centrifugation at 3000× *g* for 5 min at RT. The particles were resuspended in 75 µL of PBS and directly analyzed on a BD Cytoflex flow cytometer (East Rutherford, NJ, USA) in plate mode at a sample rate of 100 µL/minute. Sample collection was stopped following the acquisition of 75 µL. Following acquisition, the resulting FCS files were analyzed in FlowJo (Tree Star, Ashland, OR, USA). Briefly, the beads were identified by gating on singlet 3.6 µm particles in log scale in the forward scatter and side scatter parameters. APC-Cy7 channel fluorescence gates were used to segregate the particles by bead identity. Geometric mean fluorescent intensity was calculated in the PE and 488 channels. Best fit power curves were generated from the Ig capture beads using the known concentration of standards on a plate-by-plate basis. This formula was applied to the MFI of the SARS-COV-2 spike particles for all samples of the corresponding assay converting MFI to ng/mL or µg/mL concentrations. These calculated values were corrected for the dilution factor.

### 2.12. Focus Reduction Neutralization Test

A focus reduction neutralization test (FRNT) was used to quantify the titer of neutralizing antibodies against SARS-CoV-2 isolate USA-WA1/2020. Vero E6 cells were grown to confluence on 96-well plates. On the day of the infection phase of the assay, serial dilutions (1:20–1:2560) of antisera were made and combined and incubated with an equal volume of viral stock, at a specified dilution for 30 min at RT, such that the final dilutions of antisera ranged from 1:40 to 1:5120. The viral stock was diluted from a concentrated working stock to produce an estimated 30 viral focal units per well. After incubation, the sera:virus mixtures were added to the wells (100 µL), and infection allowed to proceed for 1 h on the Vero E6 cells at 35 °C. At the completion of the 1-h incubation, a viscous overlay of Eagle’s MEM with 4% FBS and antibiotics and 1.2% Avicell were added to sera:virus mixture on the cell monolayers such that the final volume was 200 µL per well. The infection was allowed to proceed for 24 h. The next day, each plate was fixed by submerging the entire plate and contents in 10% formalin/PBS for 24 h. Detection of virus foci reduction was performed on the fixed 96-well plates. Briefly, plates were rinsed in H_2_O, and methanol:hydrogen peroxide added to the wells for 30 min with rocking to quench endogenous peroxidase activity. After quenching, plates were rinsed in H_2_O to remove methanol and 5% Blotto was added to the wells as a blocking solution for 1 h. For primary antibody detection, a SARS-CoV-2 spike/RBD antibody (Sino Biological, Wayne, PA, USA) was added to 5% Blotto and incubated on the monolayers overnight. Plates were washed five times with PBS, and further incubated with a goat anti-rabbit IgG conjugated to horseradish peroxidase (Boster Bio, Pleasanton, CA, USA) in 5% Blotto for 1 h. Plates were rinsed once with 0.05% Tween in 1× PBS followed by 5 washes in 1× PBS. Impact DAB detection kit (Vector Labs, Burlingame, CA, USA) was used to detect peroxidase activity. Brown foci were counted manually from the scanned image of each well, recorded, and the reduction of foci as compared to equivalent naïve mouse sera controls was determined. FRNT_50_ titers were calculated using a 4PL curve fit to determine the serum dilution corresponding to a 50% reduction in the foci present in control wells.

### 2.13. Flow Cytometry Analysis of Innate and Adaptive Immune Cells

Cell numbers per tissue were determined by mixing 20 µL of each single-cell suspension into a 96-well plate with 50 µL of 8.4 × 10^4^ Fluoresbrite Carboxylate YG 10 µm microspheres/mL (Polysciences Inc., Warrington, PA, USA) and 180 µL staining media (dPBS + 2% FBS) containing 2 mM EDTA (SME) and 7-AAD (1:720 dilution). To perform flow cytometric analysis, 200 µL of each sample were placed into 3 separate V-bottom 96-well plates for antibody staining for flow cytometric analysis. Samples were incubated for 10 min at 4 °C in the dark with Fc-Block (clone 24G2, 10 µg/mL), washed with 200 µL staining media (SME) and then stained with myeloid, B cell, or BAL antibody panels. The myeloid panel consisted of B220/CD45R-FITC (clone RA3-6B2; 1:200 dilution), Ly6G-PE (clone 1A8; 1:200 dilution), CD64-PerCP-Cy5.5 (clone X54-5/7.1; 1:150 dilution), CD11b-APC (clone M1/70; 1:200 dilution), CD11c-PE-Cy7 (clone N418; 1:300 dilution), Ly6C-APC-Cy7 (clone AL-21; 1:200 dilution), MHCII-PB (clone M5/114.15.4; 1:600 dilution) and Aqua LIVE/DEAD (1:1000 dilution). Cells stained with the myeloid panel were incubated with the antibody mix (50 µL total volume) for 20 min at 4 °C in the dark. The B cell panel consisted of CD95/FAS-FITC (clone Jo2; 1:200 dilution), F4/80-PerCP-Cy5.5 (clone BM8; 1:200 dilution), CD3-PerCP-Cy5.5 (clone 17A2; 1:200 dilution), CD38-PE-Cy7 (clone 90; 1:400 dilution), CD19-APC-Fire750 (clone 6D5; 1:200 dilution), CD138-BV421 (clone 281-2; 1:200 dilution) and IgD-BV510 (clone 11-26c. 2a; 1:500 dilution). Cells stained with the B cell panel were incubated with antibody mix (50 µL total volume) for 45 min at 4 °C in the dark. The BAL panel consisted of Ly6G-PE (clone 1A8; 1:200 dilution), CD64-PerCP-Cy5.5 (clone X54-5/7.1; 1:150 dilution), CD8a-APC (clone 53-6.7; 1:200 dilution), CD11c-PE-Cy7 (clone N418; 1:200 dilution), CD19-APC/Fire750 (clone 6D5; 1:200 dilution; CD4-eFLUOR450 (clone GK1.5; 1:200 dilution) and Aqua LIVE/DEAD (1:1000 dilution). Cells stained with the BAL panel were incubated with the antibody mix (50 µL total volume) for 20 min at 4 °C in the dark. Following incubation with the different flow cytometry panels, the cells were washed, resuspended in 100 µL of eBioscience fixation buffer and incubated for 20 min at 4 °C. After incubation, the cells were spun down and resuspended in 200 µL SME. Stained and fixed cells from all antibody panels and cell counting panels were analyzed on a BD FACSCanto II (East Rutherford, NJ, USA). Cellular markers are summarized in Table S1. The gating strategies for T cells (Figure S7), B cells (Figure S8), myeloid cells (Figure S9), Trm cells (Figure S10) and BAL cells (Figure S11) are presented.

### 2.14. Intracellular Cytokine Staining

The analysis of CD4^+^ and CD8^+^ T cell responses in lung tissues and spleens by flow cytometry was performed as follows. Spleen and lung single-cell suspensions were stimulated with the RBD peptide pool for 5 h in the presence of 12.5 µg/mL Brefeldin A (BD Biosciences, East Rutherford, NJ, USA). Cells were then incubated on ice with a combination of fluorescent dye-labelled antibodies including anti-CD4-V500 (clone GK1.5; 1:200 dilution), anti-CD8α-APC-Fire750 (clone 53-6.7; 1:200 dilution), anti-CD11a/CD18-Pacific Blue (H155-78; 1:200 dilution), anti-CD103-PE (M290; 1:200 dilution), anti-CD69-FITC (H1-2F3; 1:200 dilution), anti-Ly6G-PerCP-Cy5.5 (clone 1A8; 1:200 dilution), anti-CD64-PerCP-Cy5.5 (clone X54-5/7.1; 1:200 dilution), anti-B220/CD45R-PerCP (clone RA3-6B2; 1:200 dilution), and Red LIVE/DEAD (1:1000 dilution). Following surface staining, cells were permeabilized using BD Biosciences Cytofix/Cytoperm kit (East Rutherford, NJ, USA) and stained with anti-IFN-γ-PE-Cy7 (XMG1.2; 1:200 dilution) and anti-TNF-α-APC (MP6-XT22; 1:200 dilution). Following incubation with the antibodies, cells were washed and resuspended before analysis on a BD FACSCanto II within 12 h.

### 2.15. IFN-γ ELISpot

Spleen and lung cell suspensions (150,000 cells/well) were placed in individual wells of ELISpot plates (Millipore-Sigma, Burlington, MA, USA) that were pre-coated with anti-IFN-γ (AN18, 5 µg/mL). Cells were stimulated with the RBD peptide pool described above at 2 µg/peptide/mL. Following 24-h stimulation, plates were stained with biotinylated anti-IFN-γ (R4-6A2), followed by washing steps, and incubation with streptavidin-AP. Secreted IFN-γ was detected following incubation with NBT/NCPI substrate for 7–10 min. The number of IFN-γ spot-forming cells were manually counted from digital images of each well.

### 2.16. Synthetic RBD Peptides

For analysis of T cell responses, a pool of 53 peptides derived from a peptide scan through the RBD of spike protein of SARS-CoV-2 (319–541) was designed and synthesized by JPT (JPT Peptide Technologies, Berlin, Germany). Peptides were designed with a length of 15 amino acids and an overlap of 11 amino acids. Before use, each vial containing 15 nM (appr. 25 µg) of each peptide per vial was reconstituted in 50 µL of DMSO before dilution into complete culture media.

### 2.17. B Cell ELISpot

Single-cell suspensions from bone marrow, BAL, and medLN cells were prepared from vaccinated mice. Cells were serially diluted in duplicate in complete media and incubated for 5 h at 37 °C on multiscreen cellulose filter ELISpot plates (Millipore-Sigma, Burlington, MA, USA) that were previously coated with purified recombinant RBD protein (Sino Biological, Wayne, PA, USA). RBD-specific antibodies secreted by plasma cells present in these tissues were detected using AP-conjugated goat anti-mouse IgG (Jackson ImmunoResearch, West Grove, PA, USA) or AP-conjugated goat anti-mouse IgA (Jackson ImmunoReserch). ELISpots were imaged and counted using S6 Ultra-V Analyzer (Cellular Technology Limited, Shaker Heights, OH, USA).

### 2.18. Measurement of Inflammatory Cytokines in Culture Supernatants

Protein levels of IFN-γ, IL-2, IL-4, IL-5, IL-10, IL-13, IL-17A, and TNF-α were quantified in culture supernatants using the mouse-specific Milliplex^®^ multi-analyte panel kit MT17MAG-47K (Millipore-Sigma, Burlington, MA, USA) and the MagPix^®^ instrument platform with related xPONENT^®^ software (Luminex Corporation, Austin, TX, USA). The readouts were analyzed with the standard version of EMD Millipore’s Milliplex^®^ Analyst software (Millipore-Sigma, Burlington, MA, USA) and VigeneTech, Inc., Carlisle, MA, USA).

### 2.19. Protective Efficacy in Mice

K18-hACE2 mice received a single dose of AdCOVID or vehicle control intranasally on day 0. On study day 28, all mice were challenged by the intranasal route with 1.4 × 10^4^ focus forming units (FFU) of SARS-CoV-2 strain 2019-nCoV/USA-AZ1/2020. Following virus challenge, body weight and survival were monitored for 24 days.

### 2.20. Quantification and Statistical Analysis

Statistical significance was assigned when *p* values were <0.05 using Prism Version 9.0.1 (GraphPad, La Jolla, CA, USA). All tests and values are indicated in the relevant figure legends.

## 3. Results

### 3.1. Intranasal Vaccination with AdCOVID Elicits Systemic and Mucosal Antibody Responses

**Figure 1:**
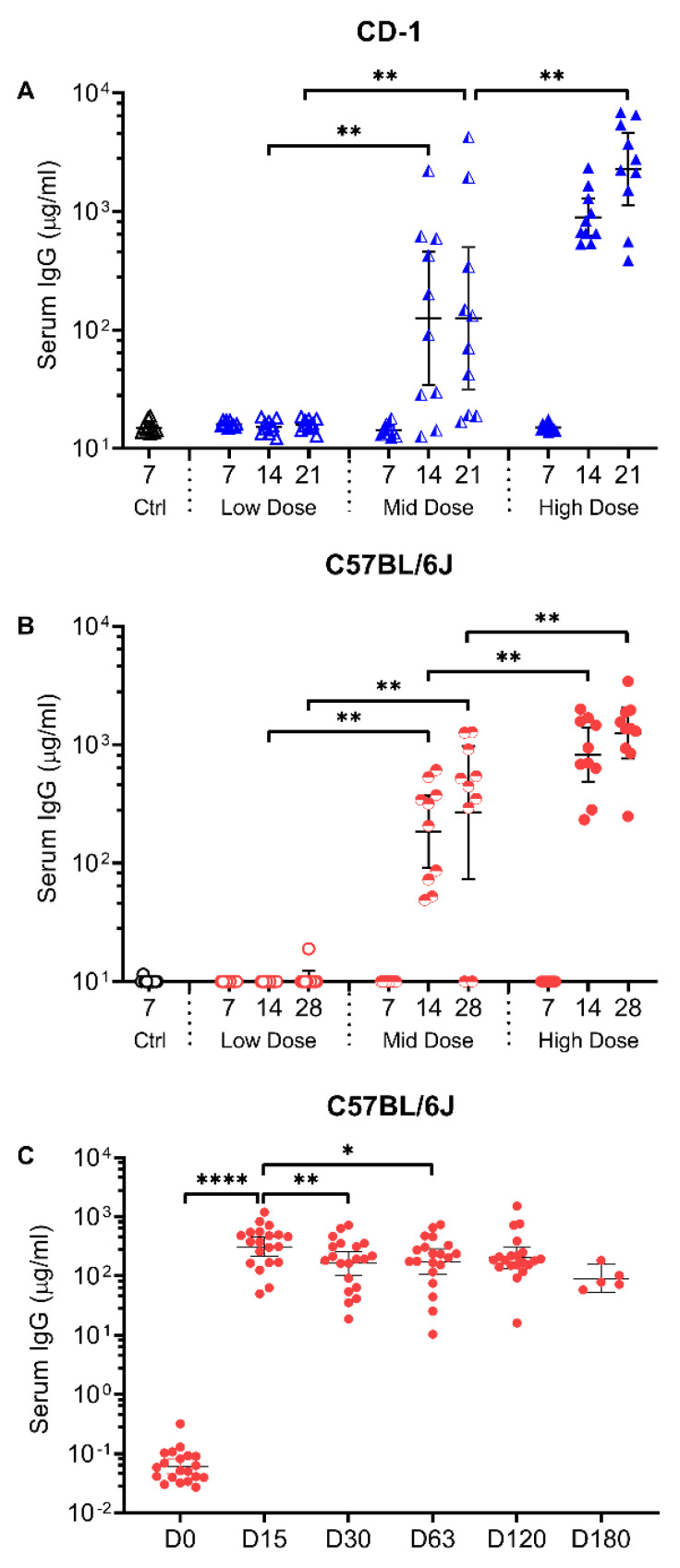
Spike-specific IgG responses in sera following a single intranasal administration of AdCOVID. (**A**) CD-1 and (**B**) C57BL/6J mice received a single intranasal administration of vehicle (Ctrl) or AdCOVID at a low, mid, or high dose as described. Sera were collected from euthanized animals between days (**A**) 7–21 or (**B**) 7–28 post-vaccination (*n* = 10 animals/group/timepoint) and analyzed individually for quantification of spike-specific IgG. (**C**) A single cohort of C57BL/6J mice (*n* = 20) received a single intranasal administration of AdCOVID at a dose of 3.78 × 10^8^ ifu. Sera were collected longitudinally between days 0–180 post-vaccination and analyzed individually for quantification of spike-specific IgG. All results are expressed in µg/mL. Data are the geometric mean response ± 95% confidence interval. Statistical analysis was performed with a Mann–Whitney test: * *p* < 0.05; ** *p* <0.01; **** *p* < 0.0001.

The intranasal COVID-19 vaccine candidate is based on a replication-deficient E1- and E3-deleted Ad5 vector platform [32]. The Ad5 vector was engineered to encode the human codon-optimized gene for the RBD (residues 302 to 543) from the spike antigen of the Wuhan-1 strain of SARS-CoV-2 (accession number QHD43416). The immunogenicity of the RBD vector (hereafter referred to as AdCOVID) was evaluated in both inbred C57BL/6J and outbred CD-1 mice. Mice received a single intranasal administration of the control vehicle or AdCOVID at 3.5 × 10^8^ infectious units (ifu) (high dose), 6 × 10^7^ ifu (mid dose) or 6 × 10^6^ ifu (low dose). No adverse effects were observed in the mice following AdCOVID vaccination. Following vaccine administration on day 0, sera and BAL samples were collected between days 7–21 (CD-1) or days 7–28 (C57BL/6J). IgG antibodies specific for SARS-CoV-2 spike were measured in sera samples using a spike cytometric bead array (CBA). Systemic spike-specific IgG antibody responses in sera were detected in both CD-1 (Figure 1A) and C57BL/6J (Figure 1B) mice following a single intranasal administration of AdCOVID. This effect appeared to be dose-dependent and notably, anti-spike IgG was detectable in sera of vaccinated mice for at least 180 days after a single intranasal dose of 3.7 × 10^8^ ifu (Figure 1C). These data are in agreement with the sustained presence of IgG-secreting, RBD-specific plasma cell populations in the mediastinal lymph node (medLN) and bone marrow of AdCOVID vaccinated mice (Figure S1).

**Figure 2:**
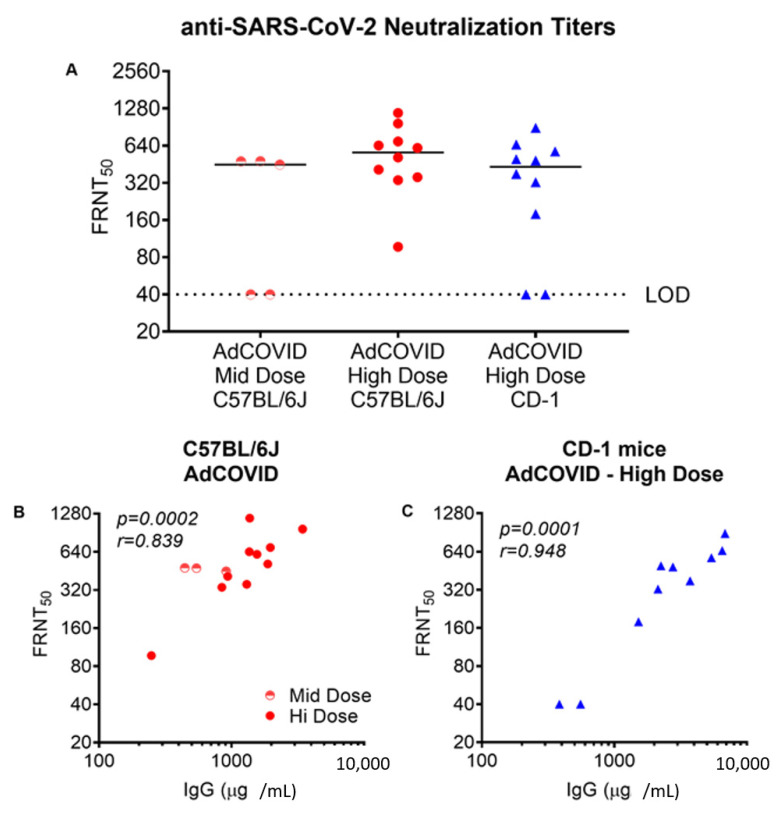
A single intranasal administration of AdCOVID elicits anti-SARS-CoV-2 neutralizing antibodies. (**A**) Neutralizing antibody response in C57BL/6J or CD-1 mice vaccinated 28 days earlier with the mid or high dose of AdCOVID as indicated. Results are expressed as the reciprocal of the dilution of serum samples required to achieve 50% neutralization (FRNT_50_) of wild-type SARS-CoV-2 infection in permissive Vero E6 cells. Data are the group median value. (**B**,**C**) Correlation between neutralizing antibody response and spike-specific IgG response in serum of vaccinated animals. Correlation analysis was performed with a two-tailed Spearman test.

The ability of AdCOVID-elicited antibodies to neutralize SARS-CoV-2 was then tested in a focus reduction neutralization test (FRNT) using the live wild-type SARS-CoV-2 isolate USA-WA1/2020. The analysis included AdCOVID samples from C57BL/6J mice 4-weeks after vaccination with either the mid (6 × 10^7^ ifu) or high dose (3.5 × 10^8^ ifu), and AdCOVID samples from CD-1 mice 3-weeks after vaccination with the high dose (3.5 × 10^8^ ifu). Intranasal AdCOVID vaccination yielded systemic neutralizing antibodies in both strains of mice (Figure 2A). At the highest AdCOVID dose, neutralizing antibody responses above background were detected in 10 of 10 C57BL/6J mice and 8 out of 10 CD-1 mice with a median titer of 563 and 431, respectively. The level of the neutralizing antibody response was well-correlated with the magnitude of the spike-specific serum IgG response measured in individual animals (Figure 2B,C), indicating that robust antibody responses to RBD were associated with generation of potentially protective neutralizing antibodies.

**Figure 3:**
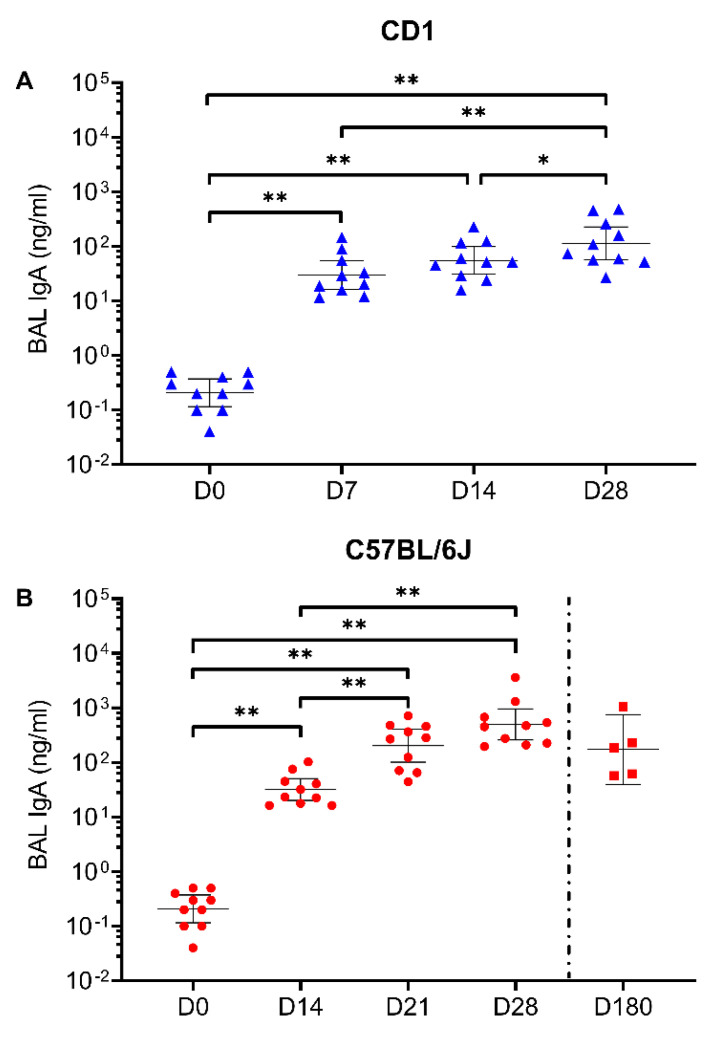
Spike-specific IgA responses in BAL following single intranasal administration of AdCOVID. (**A**) CD-1 mice (*n* = 10 animals/timepoint) received a single intranasal administration of 6.25 × 10^8^ ifu AdCOVID. BAL samples were collected at the indicated timepoints and analyzed individually for the quantification of spike-specific IgA. (**B**) C57BL/6J mice received a single intranasal administration of 6.25 × 10^8^ ifu AdCOVID. BAL samples were collected on days 0, 14, 21 and 28 (*n* = 10 animals/timepoint). In a separate study, C57BL/6J mice (*n* = 5) received a single intranasal administration of 3.78 × 10^8^ ifu AdCOVID on day 0 and were euthanized on day 180 for BAL collection. BAL samples were analyzed individually for the quantification of spike-specific IgA. All results are expressed in ng/mL. Data are the geometric mean response ± 95% confidence interval. Statistical analysis was performed with a Mann–Whitney test: * *p* <0.05; ** *p* <0.01.

IgA-mediated immunity is expected to be critical for controlling SARS-CoV-2 infection [36]. We therefore quantified the presence of anti-spike IgA in the BAL of CD-1 and C57BL/6J mice following a single intranasal dose of 6.2 × 10^8^ ifu AdCOVID. As shown in Figure 3, AdCOVID induced a lung mucosal spike-specific IgA response in both strains of mice. As was previously measured in the sera, a single intranasal administration (3.78 × 10^8^ ifu) of AdCOVID yielded long-lasting mucosal immunity with anti-spike specific IgA remaining at high levels 180 days post-vaccination (Figure 3B). These mice also had RBD-specific IgA-secreting plasma cells in both the BAL and medLN, with a preponderance of the IgA-secreting cells in the BAL (Figure S2). When combined with the presence of anti-spike IgG in the sera at day 180, these data suggest that AdCOVID elicits robust and long-lived humoral responses in both the respiratory mucosa and the periphery.

### 3.2. Intranasal AdCOVID Administration Recruits Innate and Adaptive Immune Cells to the Respiratory Tract

**Figure 4:**
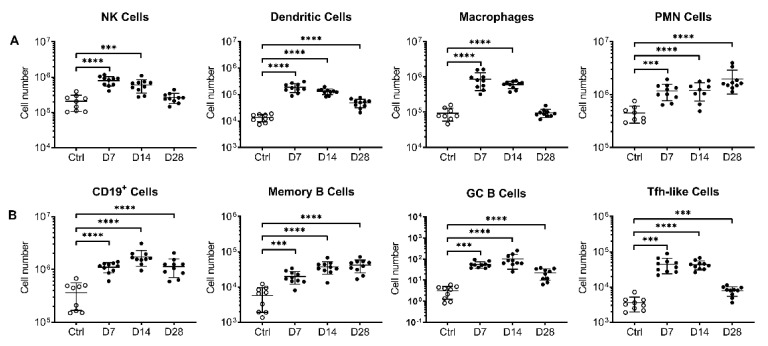
Flow cytometry analysis of immune cells in lungs from C57BL/6J mice following a single intranasal dose of AdCOVID. C57BL/6J mice were given a single intranasal administration of vehicle (Ctrl) or 3.35 × 10^8^ ifu AdCOVID. Lung cells were isolated from the vaccinated mice at the timepoints indicated (10 mice/timepoint) and analyzed individually by flow cytometry using markers of (**A**) innate immune cells or (**B**) B and Tfh-like cells as described in the Materials and Methods. Results are expressed as cell number. Different *Y*-axis scales are used across the graphics. Data are the mean response ± SD. Statistical analysis was performed with a Mann–Whitney test: *** *p* < 0.001; **** *p* < 0.0001.

Given the potent neutralizing antibody titers measured in AdCOVID vaccinated mice, we next evaluated the ability of AdCOVID to elicit cellular immunity. Flow cytometric analysis of immune cells (Table S1) was performed on lung, BAL, medLN, and spleen samples following intranasal administration of AdCOVID (3.35 × 10^8^ ifu) in C57BL/6J mice. Consistent with the hypothesis that mucosal administration of the vaccine would induce innate and adaptive respiratory immune responses, rapid recruitment of immune cells into the lung was observed following AdCOVID administration. Indicative of early innate immune activation, significant increases in the number of dendritic cells, macrophages, polymorphonuclear (PMN) cells, and natural killer (NK) cells were observed compared to control mice (Figure 4A). These responses peaked at day 7 post-vaccination. Rapid recruitment of adaptive immune cells to the lung, including T follicular helper-like cells (Tfh-like) and multiple B lineage subsets, was also observed peaking between days 7–28 (Figure 4B). Similar trends were observed in the BAL (Figure S3) and medLN (Figure S4) but were less obvious in the spleen (Figure S5).

### 3.3. AdCOVID Elicits Mucosal and Systemic RBD-Specific CD4^+^ and CD8^+^ T Cell Responses

**Figure 5:**
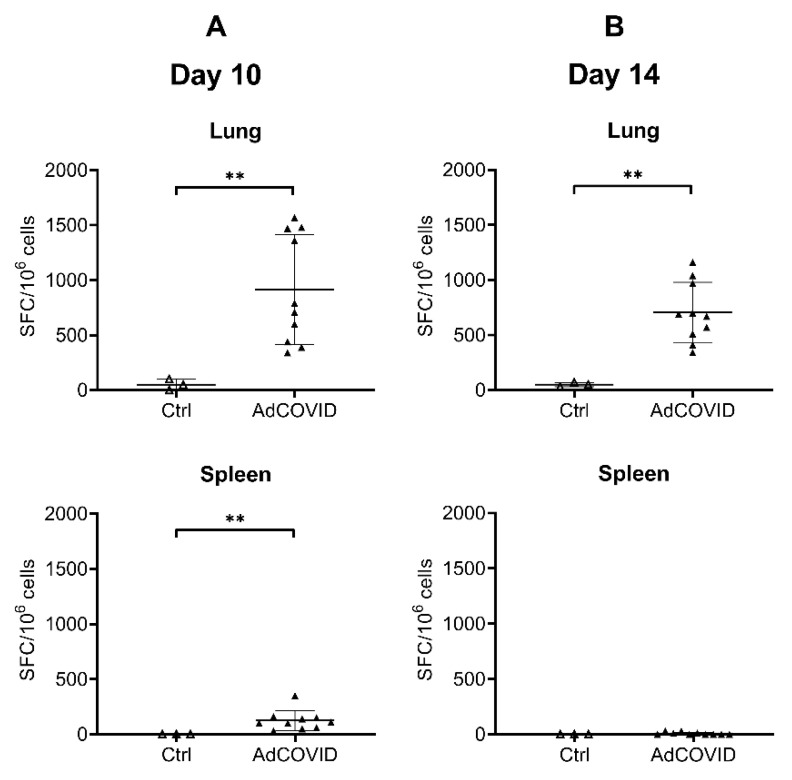
Intranasal AdCOVID vaccination elicits mucosal and systemic IFN-γ^+^ T cells. CD-1 mice were given a single intranasal administration of vehicle (Ctrl) or 3.78 × 10^8^ ifu AdCOVID. Lung and spleen cells were isolated on days (**A**) 10 and (**B**) 14 following vaccination, re-stimulated with an RBD peptide pool, and analyzed by IFN-γ ELISpot. Results are expressed as Spot Forming Cells (SFC) per million input cells. Data are the mean response ± SD. Statistical analysis was performed with a Mann–Whitney test: ** *p* < 0.01.

Animal models have shown that CD4^+^ and CD8^+^ T cells residing in the respiratory tract are important for local protection immediately after viral infection [37,38,39]. Moreover, emerging data suggest a critical role for T cell responses in COVID-19 immunity independent of antibody responses [40,41,42], particularly when directed against the SARS-CoV-2 RBD [43]. To assess vaccine-induced SARS-CoV-2-specific T cell responses, AdCOVID was administered intranasally to outbred CD-1 mice at a single dose of 3.78 × 10^8^ ifu. Control animals received vehicle alone administered intranasally. RBD-specific T cell cytokine responses in lung and spleen samples were assessed by ELISpot following ex vivo re-stimulation with a pool of 54 peptides (15 amino acids long, 11 amino acid overlap) covering the SARS-CoV-2 RBD residues 319–541. A high frequency of RBD-specific IFN-γ^+^ T cells were detected in the lung at 10- (Figure 5A) and 14- (Figure 5B) days post-vaccination, reaching a mean response of 915 and 706 spot forming cells (SFC) per million input cells respectively. IFN-γ producing, RBD-specific T cells were also detected by ELISpot in the spleen (Figure 5A,B), albeit at lower frequency compared to the lung. These results provide evidence that mucosally-delivered vaccines can elicit functional effector T cells that are distributed in secondary lymphoid tissues.

**Figure 6:**
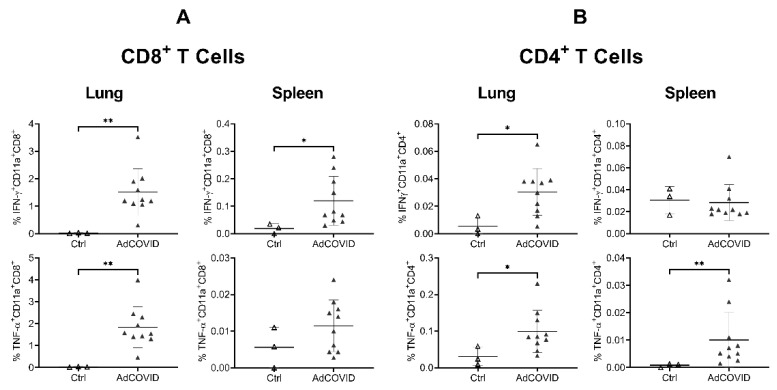
Intracellular cytokine production by pulmonary and splenic T cells 14 days after intranasal AdCOVID vaccination. CD-1 mice were given a single intranasal administration of vehicle (Ctrl) or 3.78 × 10^8^ ifu AdCOVID. Lung cells (*n* = 10 mice/vaccine, 3 mice/control) were isolated on day 14, re-stimulated with the RBD peptide pool for 5 h, and analyzed by flow cytometry. Results are expressed as the percentage of IFN-γ or TNF-α expressing (**A**) CD11a^+^CD8^+^ or (**B**) CD11a^+^CD4^+^ T cells for individual mice. Different *Y*-axis scales are used across the graphics. Data are the mean response ± SD. Statistical analysis was performed with a Mann–Whitney test: * *p* < 0.05; ** *p* < 0.01.

**Figure 7:**
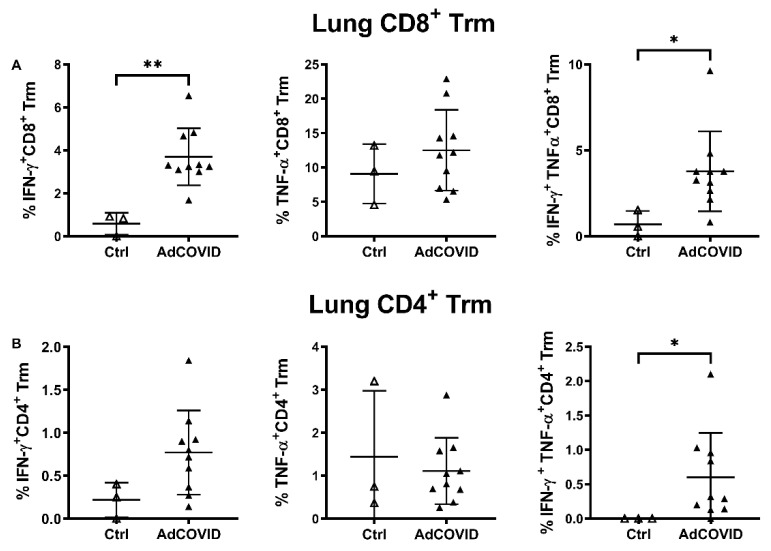
Intranasal AdCOVID vaccination elicits polyfunctional memory T cell populations in the lung 14 days after vaccination. CD-1 mice were given a single intranasal administration of vehicle (Ctrl) or 3.78 × 10^8^ ifu AdCOVID. Lung cells (*n* = 10 mice/vaccine, 3 mice/control) were isolated at day 14, re-stimulated with the RBD peptide pool for 5 h, and analyzed by flow cytometry to identify CD69^+^CD103^+^ resident memory T cells (Trm). Results are expressed as the percentage of IFN-γ^+^, TNF-α^+^, or double positive IFN-γ^+^/TNF-α^+^ expressing (**A**) CD8^+^ or (**B**) CD4^+^ Trm cells for individual mice. Different *Y*-axis scales are used across the graphics. Data are the mean response ± SD for the groups. Statistical analysis was performed with a Mann–Whitney test: * *p* < 0.05; ** *p* < 0.01.

To further characterize the RBD-specific CD4^+^ and CD8^+^ T cell responses to mucosal vaccination, intracellular cytokine staining was performed on lung and spleen cells from vaccinated CD-1 mice (3.78 × 10^8^ ifu). Consistent with ELISpot data, we observed a significant induction of IFN-γ- or TNF-α-producing T cells in the lung and spleen of vaccinated animals. These included both CD11a^+^CD8^+^ (Figure 6A) and CD11a^+^CD4^+^ (Figure 6B) T cells, although the response was strongly biased toward the induction of CD8^+^ T cells, especially in lung samples. Expression of integrin CD11a, which is only upregulated in recently activated T cells and is required for optimal vascular adhesion in the tissue and retention within the respiratory tract [44], supports the hypothesis that these cells were recently recruited to the lung. To assess whether these cells are resident memory T cells (Trm), the expression of the Trm markers CD103 and CD69 on pulmonary CD4^+^ and CD8^+^ cells was measured [39]. Consistent with the intranasal administration route, induction of lung RBD-specific CD8^+^ and CD4^+^ Trm expressing either IFN-γ, TNF-α or both cytokines was observed (Figure 7).

### 3.4. Intranasal AdCOVID Vaccination Yields a Type 1 Cytokine-Biased Immune Response

A vaccine-elicited T_h_2 biased immune response may result in VAED following SARS-CoV-2 infection. Our data showed that intranasal administration of AdCOVID induced both monofunctional and polyfunctional T cells capable of producing type 1-associated cytokines, such as IFN-γ and TNF-α. In addition, the vaccine elicited high frequencies of antigen-specific CD8^+^ T cells that generally correlate with an interferon-regulated T cell response necessary for control of viral infection. To further assess the cytokine-producing potential of T cells from AdCOVID vaccinated CD-1 mice (3.78 × 10^8^ ifu), splenic T cells were stimulated with RBD peptides for 48 h and then the presence of secreted cytokines was measured in harvested supernatant by multiplex cytokine bead array (Figure S6). As expected, induction of IFN-γ and TNF-α by T cells was observed. Moreover, T cells from the vaccinated animals produced moderate levels of IL-10 compared to T cells from the vehicle control-treated mice. Importantly, the levels of the T_h_2 and T_h_17-derived cytokines, including Interleukin (IL)-4, IL-5, IL-13 and IL-17A, were statistically equivalent in the supernatants of peptide-stimulated cells isolated from the spleens of vaccinated and control animals. These data therefore indicated that intranasal administration of AdCOVID did not potentiate a deleterious T_h_2 response but rather induced the expected antiviral T cell responses.

### 3.5. Intranasal AdCOVID Vaccination Protects against Lethal SARS-CoV-2 Challenge

**Figure 8:**
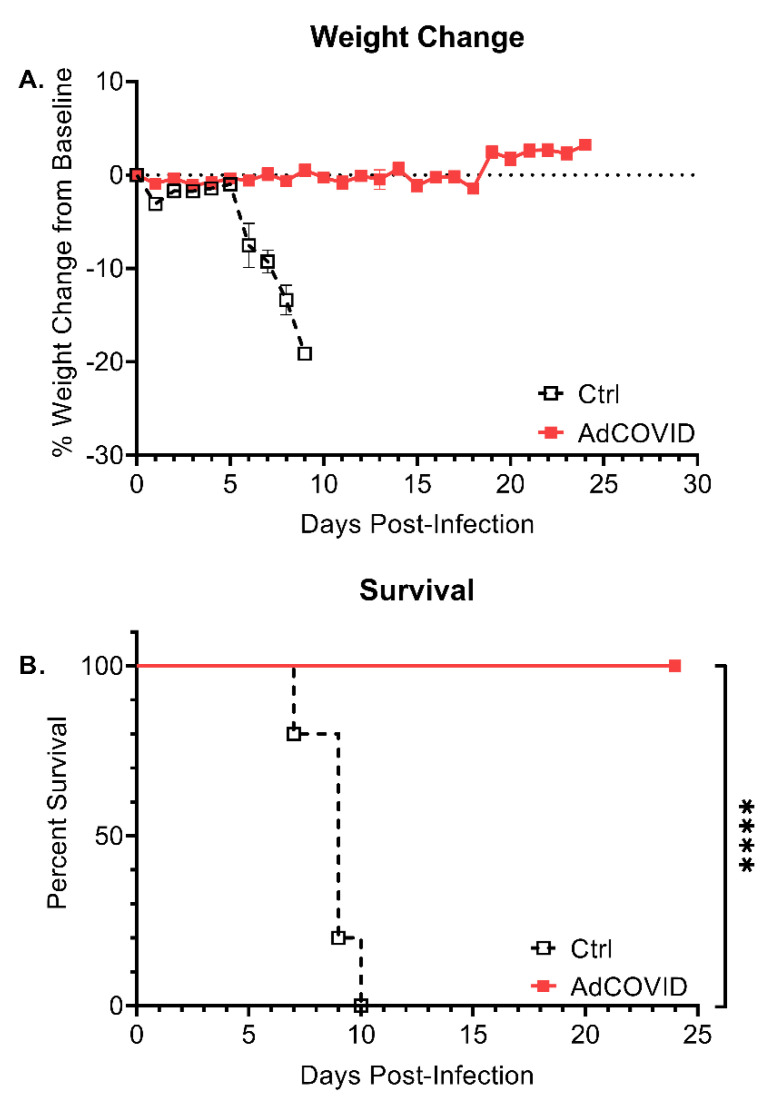
A single intranasal dose of AdCOVID completely protects K18-hACE2 mice from lethal SARS-CoV-2 challenge. K18-hACE2 mice were given a single intranasal administration of vehicle (Ctrl, *n* = 5) or AdCOVID (6 × 10^8^ ifu, *n* = 10). All mice were intranasally challenged 28 days post-vaccination with 1.4 × 10^4^ FFU of 2019-nCoV/USA-AZ1/2020 and monitored for change of body weight and mortality for 24 days. (**A**) Percent weight change from baseline and (**B**) group survival of 2019-nCoV/USA-AZ1/2020 challenged mice. Weight loss is presented as the mean ± SEM. Survival data is presented as Kaplan–Meier survival curves and analysis was performed by the Log-rank (Mantel–Cox). **** *p* < 0.0001.

We next tested the protective efficacy of AdCOVID in the widely used K18-hACE2 mouse model [45]. K18-hACE2 mice received a single intranasal administration of vehicle control or AdCOVID (6 × 10^8^ ifu). 28 days post-vaccination, all mice were challenged intranasally with 1.4 × 10^4^ FFU of SARS-CoV-2 isolate 2019-nCoV/USA-AZ1/2020. Animal weights and survival were followed until study termination 24 days post-infection (DPI). Following challenge, 5/5 vehicle control mice exhibited significant weight loss beginning at DPI +5 (Figure 8A) and succumbed to COVID-19-like disease by DPI +10 (Figure 8B). Notably, 10/10 AdCOVID vaccinated mice survived SARS-CoV-2 challenge through DPI +24 (Figure 8B) in the absence of significant weight loss (Figure 8A).

## 4. Discussion

To date, the COVID-19 vaccine candidates that have achieved emergency use approval or that have advanced to Phase 3 clinical trials are delivered by intramuscular injection. In preclinical and clinical studies, these candidates have demonstrated stimulation of serum neutralizing antibodies and peripheral T cell responses. However, a fundamental limitation of these approaches is that they are not designed to target the distinct immune microenvironment of the respiratory tract mucosa, and there is no widespread expectation that the current intramuscular vaccine candidates will provide sterilizing immunity in these spaces. In a SARS-CoV-2 challenge model in rhesus macaques, a single intramuscular administration of an adenovectored vaccine candidate was shown to significantly reduce viral load in the bronchoalveolar lavage fluid and lower respiratory tract tissue but the level of viral replication in the nasal cavity was unaffected [46]. Conversely, nasal administration of replication-deficient human Ad5-vectored vaccines, such as AdCOVID, mimic natural infection of respiratory viruses and stimulate strong protective immunity, both systemically and mucosally, while maintaining an established clinical safety profile [47,48,49,50,51]. Intranasal vaccination stimulates mucosal IgA antibodies, providing a first line of defense at the point of respiratory pathogen inoculation [52], which correlates well with protection from respiratory infections such as influenza [53,54,55]. Numerous studies have demonstrated the ability of intranasally administered vaccines to block transmission of influenza between infected and naïve cage-mates [56,57]. Moreover, intranasal administration bypasses preexisting immunity to the adenovector [47]. Consistent with these reports, several studies assessing other COVID-19 vaccine candidates have shown that the intranasal route, as opposed to the intramuscular route, stimulated local mucosal immune responses in addition to systemic neutralizing antibody and T cell responses, resulting in significantly reduced oropharyngeal virus shedding compared to intramuscularly vaccinated animals [23,58,59].

In agreement with these reports, we showed here that AdCOVID, an intranasal adenovirus-vectored vaccine encoding the RBD of the SARS-CoV-2 spike protein, elicited robust systemic and local immunity. One intranasal dose of AdCOVID resulted in a focused immune response against SARS-CoV-2 spike antigen, including the induction of functional serum antibodies that neutralized wild-type SARS-CoV-2 virus. Importantly, AdCOVID vaccination also elicited antigen-specific IgA, IgG, and polyfunctional CD8^+^ and CD4^+^ T cell responses in the respiratory tract, suggesting balanced mucosal immunity. Cell-mediated responses induced by AdCOVID were biased toward antiviral type 1 cytokine responses as demonstrated by the high rates of antigen-specific CD8^+^ T cells and an effector cytokine profile including IFN-γ and TNF-α. Most notably, a single intranasal administration of AdCOVID completely protected K18-hACE2 mice from morbidity and mortality following lethal SARS-CoV-2 challenge.

Key to these findings, intranasal AdCOVID vaccination appears to yield a significant memory response, both locally and systemically. AdCOVID promoted persistent spike-specific sera IgG responses with no evidence of significant decline six months after a single vaccination. Likewise, IgA levels in the respiratory tract remained elevated throughout the course of the study. When combined with the establishment of a resident memory CD8^+^ T cell population in the lungs, our data suggest that AdCOVID has the potential to confer long lasting protective immunity, particularly within the bronchoalveolar space and lungs, which represent major sites of infection and clinical disease.

By what mechanism was AdCOVID able to elicit such robust memory responses? One possibility is that the immune response induced by AdCOVID vaccination was targeted to the RBD domain alone, which contains the critical neutralizing epitopes in the spike protein. This is consistent with results obtained during the development of vaccines for SARS-CoV [60]. We postulate that an Ad5-vector expressing RBD vaccine may offer three advantages over other forms of the spike antigen that have been used in COVID-19 vaccine candidates currently in clinical development. First, it is reported that RBD-based vaccines can promote equivalent or higher titer antibody responses than the full-length or S1/S2 ectodomain of the SARS-CoV-2 spike [60,61]. The improved affinity and neutralizing activity of RBD-based vaccine-elicited antibodies is attributed to immunofocusing [62,63]. Second, this permits not only targeting of the receptor-binding motif (RBM), but also targeting of more conserved subdominant or cryptic epitopes within the RBD core; thereby lessening the effect of RBD point mutations and allowing for potent cross-variant neutralization [64,65]. Third, recent data suggest that neutralizing antibody responses directed against the RBD are less impacted by escape mutations compared to neutralizing antibodies that target other regions of the spike protein [66]. These factors suggest that RBD-only vaccines, such as AdCOVID, have the potential to provide significant protection from multiple SARS-CoV-2 variants while avoiding immune erosion by viral evolution.

In summary, there is a clear need for a vaccine to prevent SARS-CoV-2 infection, preferably one that elicits mucosal immunity so as to reduce viral shedding in the upper respiratory tract, thereby reducing transmission. In these experiments, AdCOVID induced systemic and mucosal immune responses within days following a single-dose vaccination. In the context of a pandemic, intranasally administered AdCOVID has two compelling advantages. First, non-invasive intranasal administration makes it particularly well-suited for widespread vaccinations of large cohorts. While intranasal vaccination with live-attenuated vaccines (LAIV) is not recommended in individuals less than two years and over fifty years of age, we do not anticipate a similar limitation would be necessary for AdCOVID. The age restrictions for LAIV stem from the replication of the LAIV in individuals with potentially compromised immune function. As AdCOVID is a replication-deficient vector, we do not expect a potent inflammatory response following administration as can occur with LAIV. This is evidenced by the waning of the inflammatory response in the lungs by day 7. Intranasal administration may also provide incentive for the subset of the population displaying hesitance towards vaccination due to trypanophobia [21]. Second, intranasal AdCOVID may control SARS-CoV-2 infection within both the upper and lower respiratory tract. This has the advantages of potentially preventing infection at the site of virus entry, reducing the risk of significant respiratory disease, and decreasing the likelihood of subsequent virus transmission by vaccinated individuals. Collectively, these findings support further development of AdCOVID for the prevention of SARS-CoV-2 infections and its transmission. These aspects will be investigated as the intranasal AdCOVID vaccine progresses through ongoing clinical trials.

## 5. Conclusions

A single intranasal dose of AdCOVID, an adenovirus type 5-vectored vaccine encoding the RBD of the SARS-CoV-2 spike protein, induced cellular and humoral immunity in the mucosa and periphery of mice that persisted for the duration of the six-month study. Mirroring the natural immune response to SARS-CoV-2 infection, AdCOVID vaccinated animals had spike-specific IgG, IgA, and T cell populations in the respiratory tract. Additionally, a single intranasal dose of AdCOVID completely protected mice from lethal challenge, preventing morbidity and mortality. These data have important implications for preventing COVID-19 disease and may provide a pathway to blocking SARS-CoV-2 transmission between hosts. Future preclinical studies will investigate the ability of AdCOVID to halt transmission as well as protect against non-vaccine-matched viral challenge.

## 6. Patents

Altimmune Inc. has filed the following patent applications related to AdCOVID: PCT/US21/17920; US 17/175,144 and US 17/175,131.

## Data Availability

The data that support the findings of this study are available from the authors on reasonable request pending approval from all relevant institutions.

## Supplementary Materials

The following are available online at https://www.mdpi.com/article/10.3390/vaccines9080881/s1, Figure S1. A single intranasal dose of AdCOVID generates long-lived anti-RBD IgG-secreting B cell populations in the periphery, Figure S2. A single intranasal dose of AdCOVID generates long-lived anti-RBD IgA-secreting B cell populations, Figure S3. Flow cytometry analysis of immune cells in BAL from C57BL/6J mice following intranasal vaccination with a single dose of AdCOVID, Figure S4. Flow cytometry analysis of immune cells in medLN nodes from C57BL/6J mice following intranasal vaccination with a single dose of AdCOVID, Figure S5. Flow cytometry analysis of immune cells in spleens from C57BL/6J mice following intranasal vaccination with a single dose of AdCOVID, Figure S6. Intranasal AdCOVID vaccination does not elicit a T_h_2 or T_h_17-biased immune response, Table S1. Flow Cytometry Cellular Markers, Figure S7. Representative flow cytometry plots for gating of T cells isolated from mice, Figure S8. Representative flow cytometry plots for gating of B cells isolated from mice, Figure S9. Representative flow cytometry plots for gating of myeloid cells isolated from mice, Figure S10. Representative flow cytometry plots for gating of lung Trm cells isolated from mice, Figure S11. Representative flow cytometry plots for gating of BAL cells isolated from mice.
