# Supplementary material for: Single-Dose Intranasal Administration of AdCOVID Elicits Systemic and Mucosal Immunity against SARS-CoV-2 and Fully Protects Mice from Lethal Challenge
Source: Vaccines (Basel). 2021 Aug 9;9(8):881. doi: 10.3390/vaccines9080881 (PMC8402488; doi:10.3390/vaccines9080881)
Supplement: Supplementary file 1 [file vaccines-09-00881-s001.zip › vaccines-1291313-supplementary.pdf]

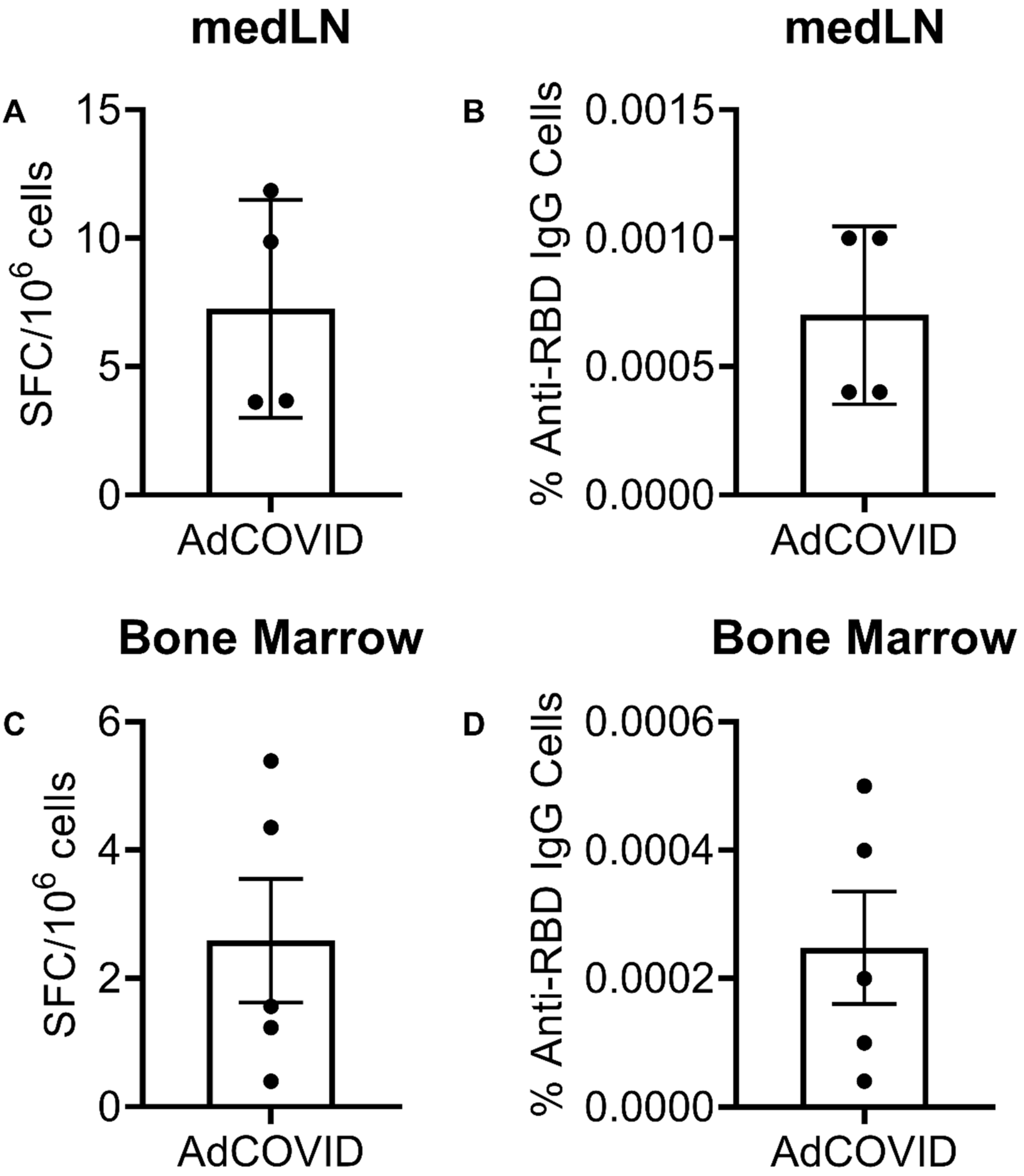

Figure S1. A single intranasal dose of AdCOVID generates long-lived anti-RBD IgG-secreting B cell populations in the periphery. C57BL/6J mice (n=10) received a single intranasal administration of AdCOVID at a dose of 3.78E+08 ifu on day 0. Mice were euthanized on day 193 and (**A, B**) medLN or (**C, D**) bone marrow were harvested for B cell ELISpot. Results are expressed as (A, C) Spot Forming Cells (SFC) per million input cells or (B, D) frequency of RBD-specific IgG-secreting cells isolated. Data are the mean response +/- SD.

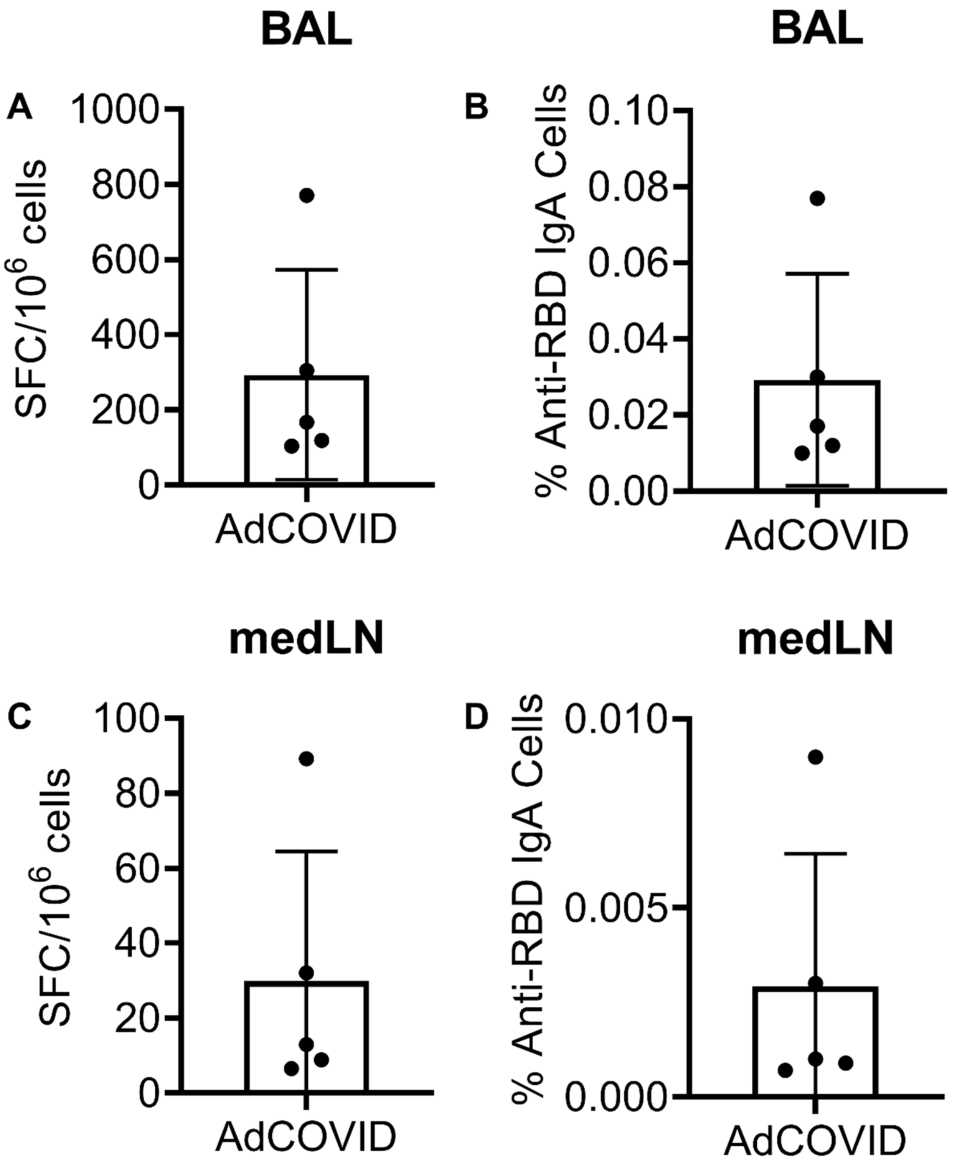

Figure S2. A single intranasal dose of AdCOVID generates long-lived anti-RBD IgA-secreting B cell populations. C57BL/6J mice (n=5) received a single intranasal administration of AdCOVID at a dose of 3.78E+08 ifu on day 0 and were euthanized on day 180. Cells were isolated from the (**A, B**) BAL and (**C, D**) medLN for analysis by B cell ELISpot. Results are expressed as (A, C) Spot Forming Cells (SFC) per million input cells or (B, D) frequency of RBD-specific IgA-secreting cells isolated. Data are the mean response +/- SD.

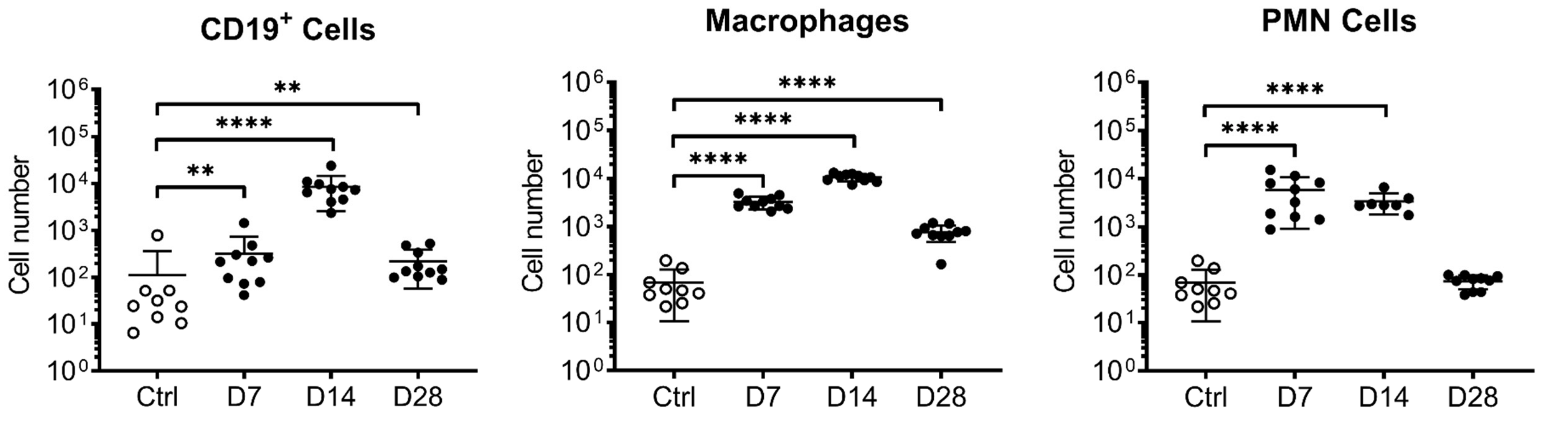

Figure S3. Flow cytometry analysis of immune cells in BAL from C57BL/6J mice following intranasal vaccination with a single dose of AdCOVID. C57BL/6J mice were given a single intranasal administration of vehicle (Ctrl) or 3.35E+08 ifu AdCOVID (high dose). BAL cells were collected from vaccinated animals at the indicated timepoints (10 mice/timepoint) and analyzed individually by flow cytometry as described in the Materials and Methods. Results are expressed as cell number. Data are mean response +/- SD. Statistical analysis was performed with a Mann-Whitney test: **, P <0.01; ****, P < 0.0001.

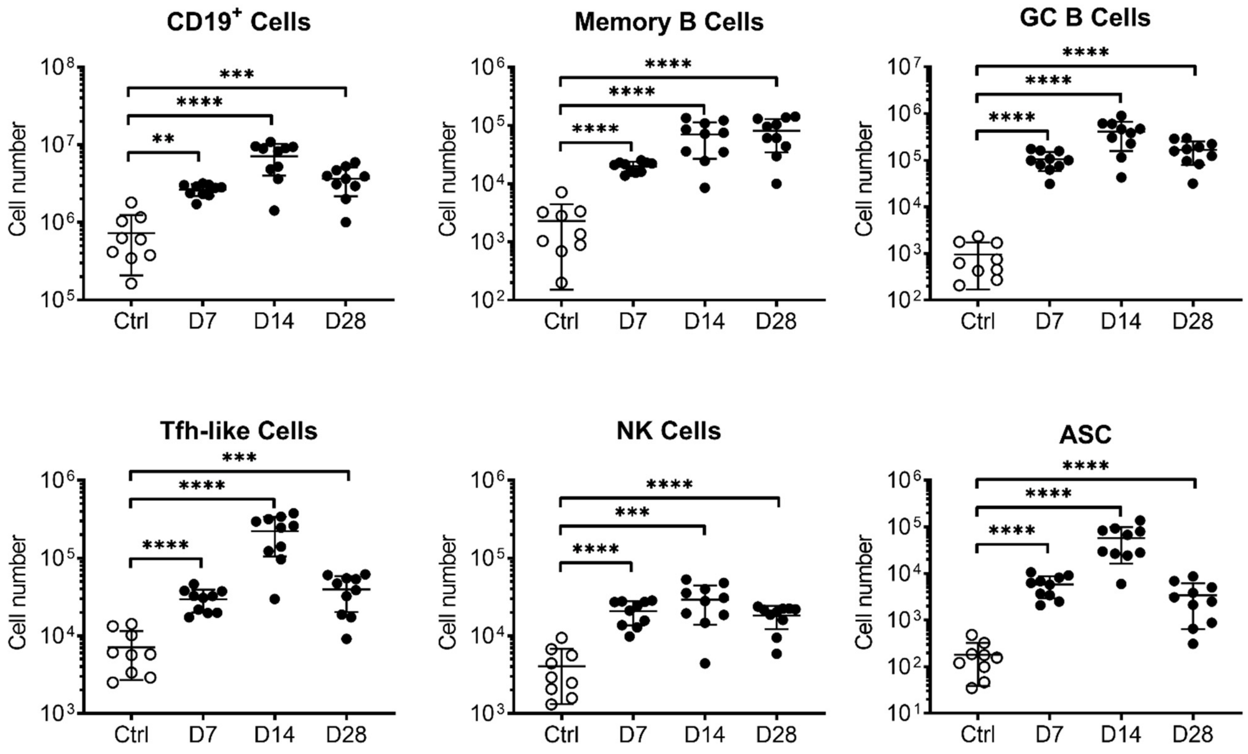

Figure S4. Flow cytometry analysis of immune cells in medLN nodes from C57BL/6J mice following intranasal vaccination with a single dose of AdCOVID. C57BL/6J mice were given a single intranasal administration of vehicle (Ctrl) or 3.35E+08 ifu AdCOVID (high dose). medLN cells were isolated from vaccinated animals at the indicated timepoints (10 mice/timepoint) and analyzed individually by flow cytometry as described in the Materials and Methods. Results are expressed as cell number. Data are mean response +/- SD. Statistical analysis was performed with Mann-Whitney test: **, $P < 0.01$; ***, $P < 0.001$; ****, $P < 0.0001$.

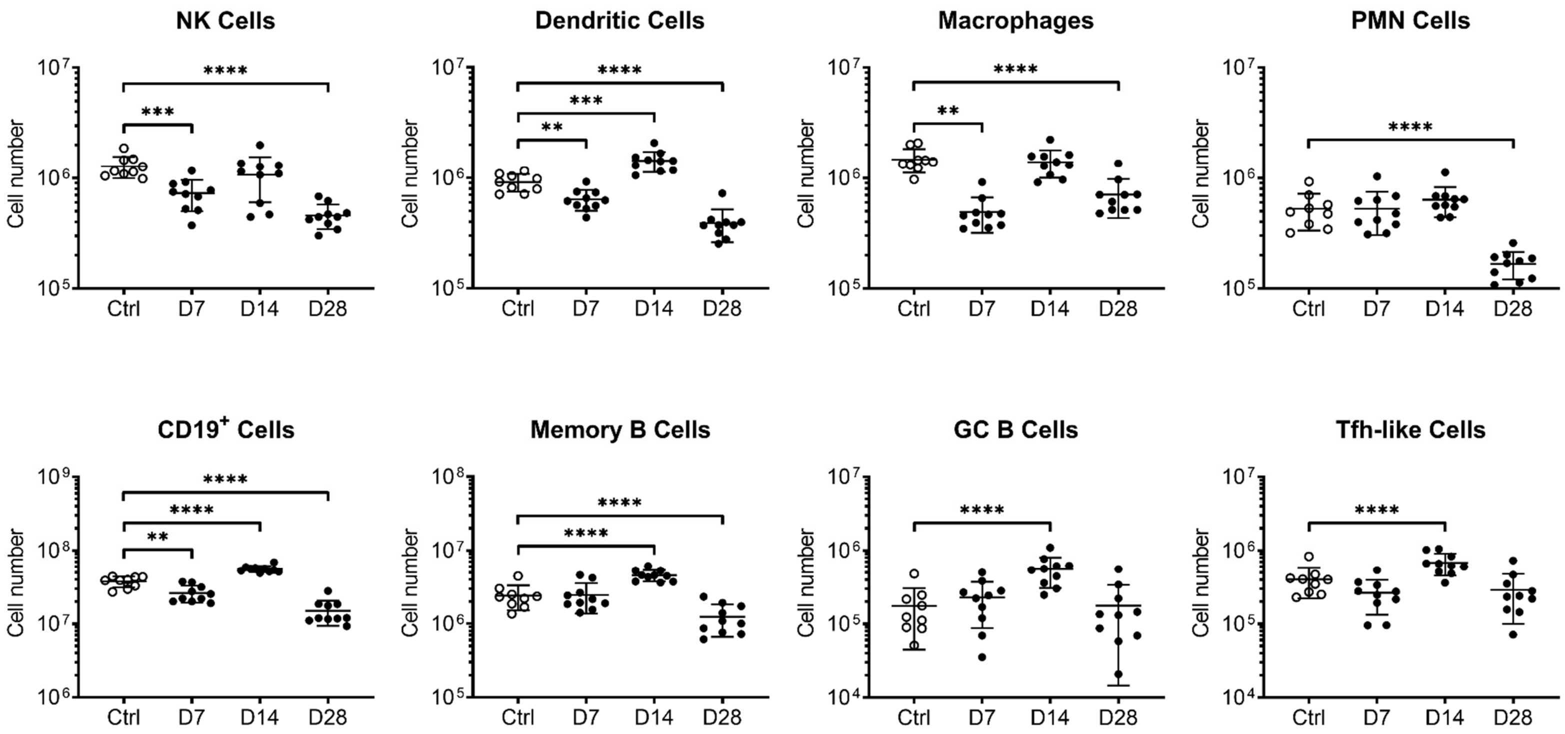

Figure S5. Flow cytometry analysis of immune cells in spleens from C57BL/6J mice following intranasal vaccination with a single dose of AdCOVID. C57BL/6J mice were given a single intranasal administration of vehicle (Ctrl) or 3.35E+08 ifu AdCOVID (high dose). Splenocytes were collected from vaccinated animals at the indicated timepoints (10 mice/timepoint) and analyzed individually by flow cytometry as described in the Materials and Methods. Results are expressed as cell number. Data are mean response +/- SD. Statistical analysis was performed with Mann-Whitney test: **, P <0.01; ***, P < 0.001; ****, P < 0.0001

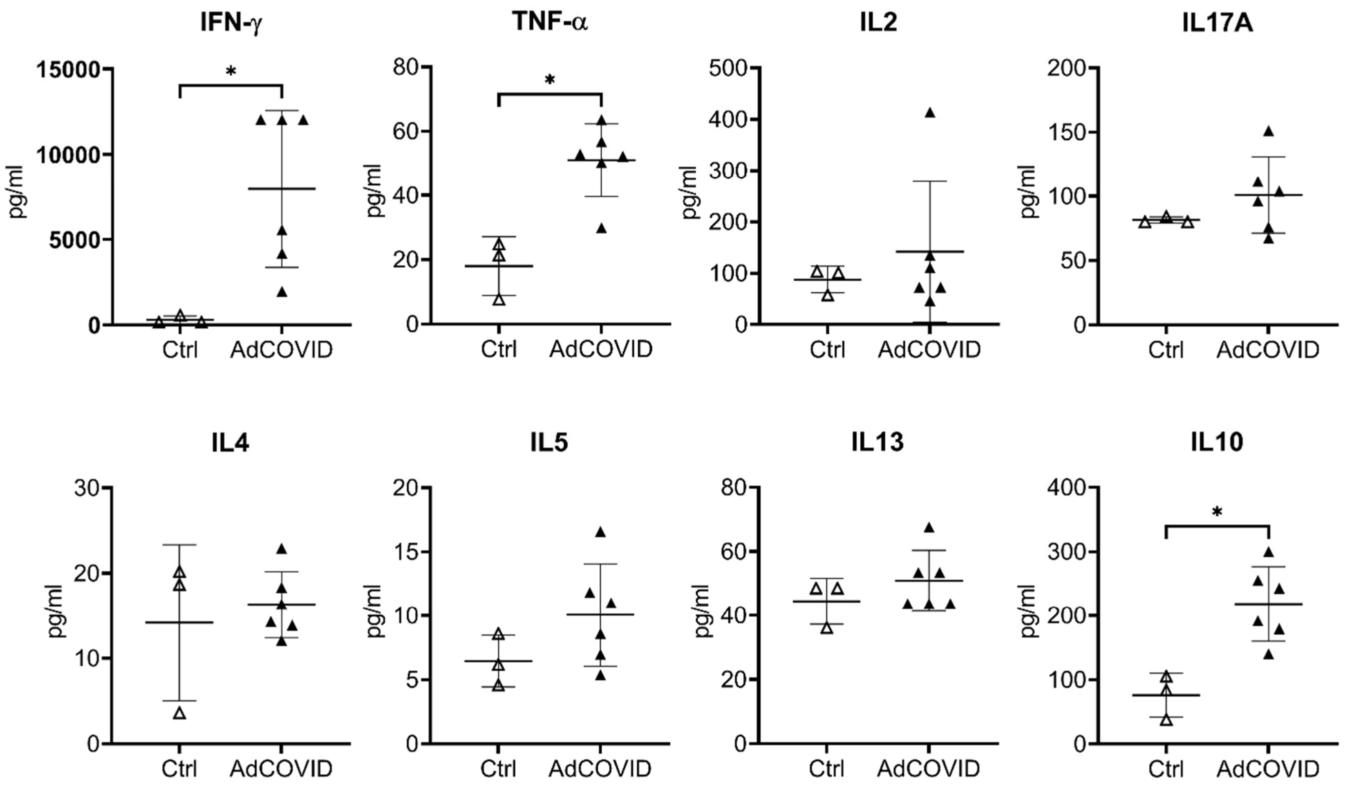

Figure S6. Intranasal AdCOVID vaccination does not elicit a T_h2 or T_h17-biased immune response. CD-1 mice were given a single intranasal administration of vehicle (Ctrl) or 3.78E+08 ifu AdCOVID. Splenocytes (n= 10 mice/vaccine, 3 mice/control) were isolated at day 10 and re-stimulated with the RBD peptide pool for 48 hours. Secreted cytokines were detected in the supernatant using a cytokine multiplex assay. Results are expressed in pg/mL. Different Y-axis scales are used across the graphics. Data are mean response +/- SD. Statistical analysis was performed with a Mann-Whitney test: *, $P < 0.05$.

Table S1. Flow Cytometry Cellular Markers

Cell Type	Cellular Markers
CD4 T Cell	$CD3^{+}CD4^{+}$
CD8 T Cell	$CD3^{+}CD8^{+}$
T Follicular Helper-like (Tfh)	$CD4^{+}CD25^{lo}CXCR5^{+}PD\text{-}1^{hi}$
CD19 B Cell	$CD19^{+}CD138^{neg}$
Memory B Cell	$CD19^{+}IgD^{neg}CD38^{+}Fas^{neg}$
Germinal Center B Cell (GC)	$CD19^{+}IgD^{neg}CD38^{lo}Fas^{+}$
Antibody Secreting Cells (ASC)	$CD19^{lo}CD38^{lo}CD138^{hi}$
Natural Killer (NK)	$CD3^{neg}NK1.1^{+}$
Polymorphonuclear (PMN)	$Ly6G^{hi}$
Macrophage	$Ly6G^{neg}CD11b^{+}CD64^{hi}$
Dendritic Cell	$Ly6H^{neg}CD64^{lo/neg}MHCII^{+}CD11c^{+}$
BAL CD19 B cell	$Autof^{neg}Ly6G^{neg}CD64^{neg}CD4^{neg}CD8^{neg}CD19^{+}$
BAL CD4 T cell	$Autof^{neg}Ly6G^{neg}CD64^{neg}CD4^{+}$
BAL CD8 T cell	$Autof^{neg}Ly6G^{neg}CD64^{neg}CD8^{+}$
Alveolar macrophage (AM)	$Autof^{+}Ly6G^{neg}CD64^{neg}CD11c^{+}$

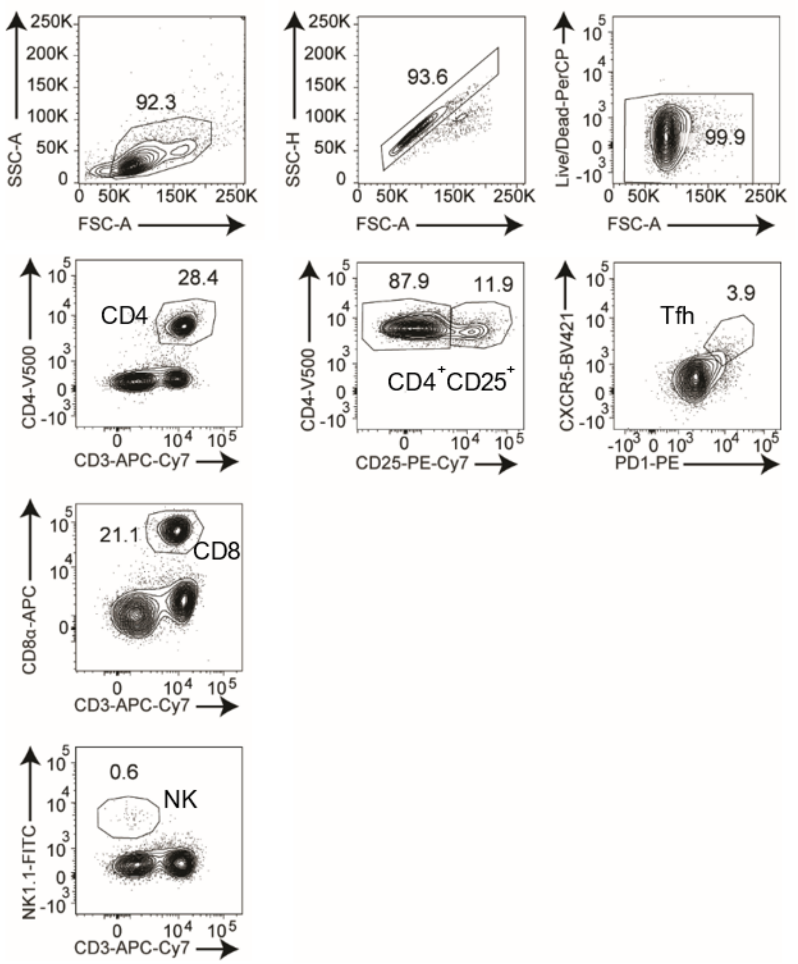

Figure S7. Representative flow cytometry plots for gating of T cells isolated from mice. Gating strategy to identify $CD3^+CD4^+$ ($CD4^+$ T cells), $CD3^+CD8^+$ ($CD8^+$ T cells), $CD3^{neg}NK1.1^+$ (NK cells), $CD25^+$ CD4 T cells and $CXCR5^+PD\text{-}1^{hi}$ T follicular helper-like cells (Tfh).

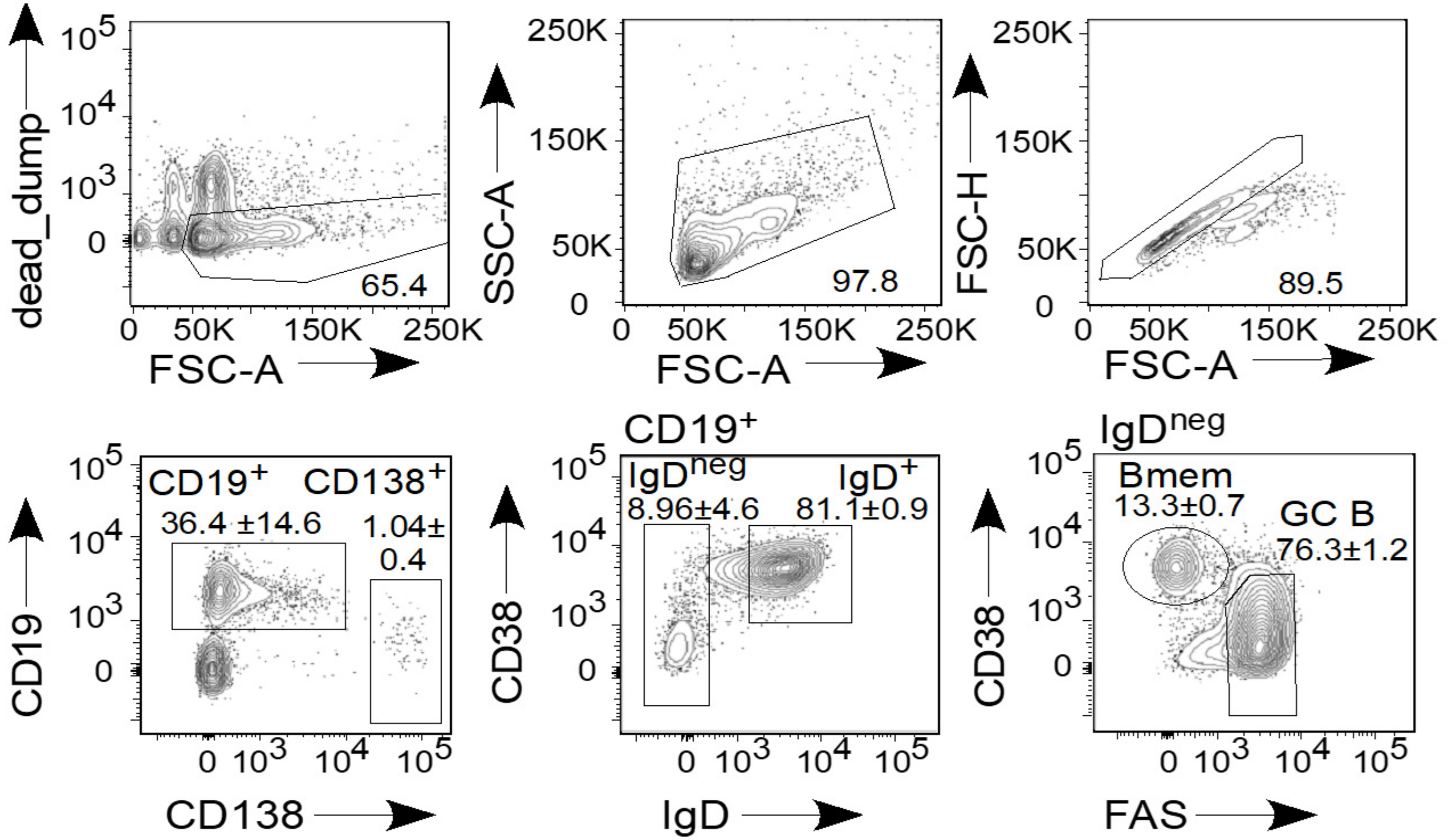

Figure S8. Representative flow cytometry plots for gating of B cells isolated from mice. Gating strategy to identify total CD19^{+} B cells, CD19^{lo}CD138^{hi} antibody secreting cells (ASCs), IgD^{+} naïve B cells, IgD^{neg} antigen-experienced B cells, IgD^{neg}CD38^{+}Fas^{neg} memory B cells (Bmem) and IgD^{neg}CD38^{lo}Fas^{+} germinal center (GC) B cells.

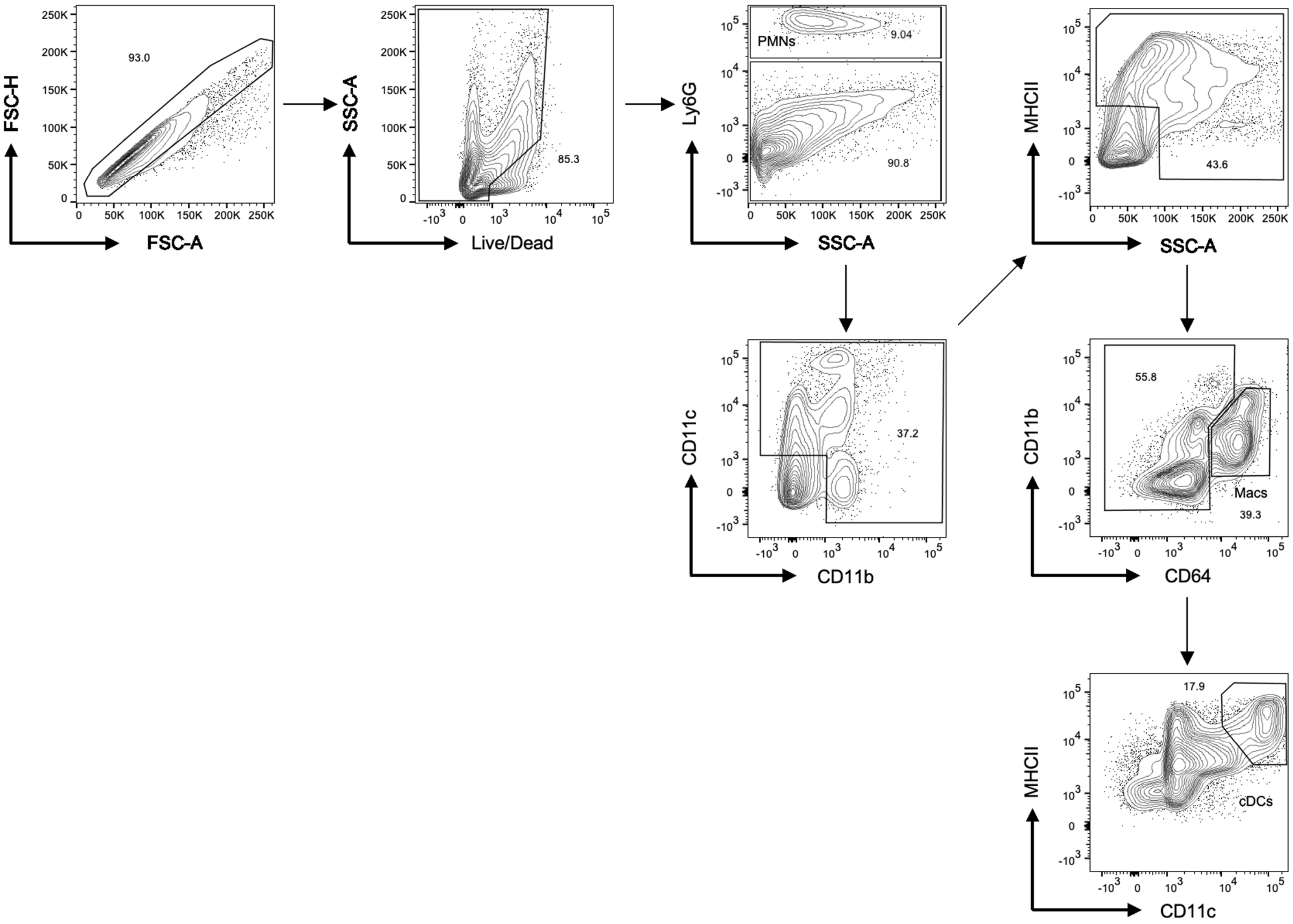

Figure S9. Representative flow cytometry plots for gating of myeloid cells isolated from mice. Gating strategy to identify Ly6G^{hi} neutrophils (PMN), CD11b^{+}CD64^{hi} macrophages, and MHCII^{+}CD11c^{+} dendritic cells (cDCs).

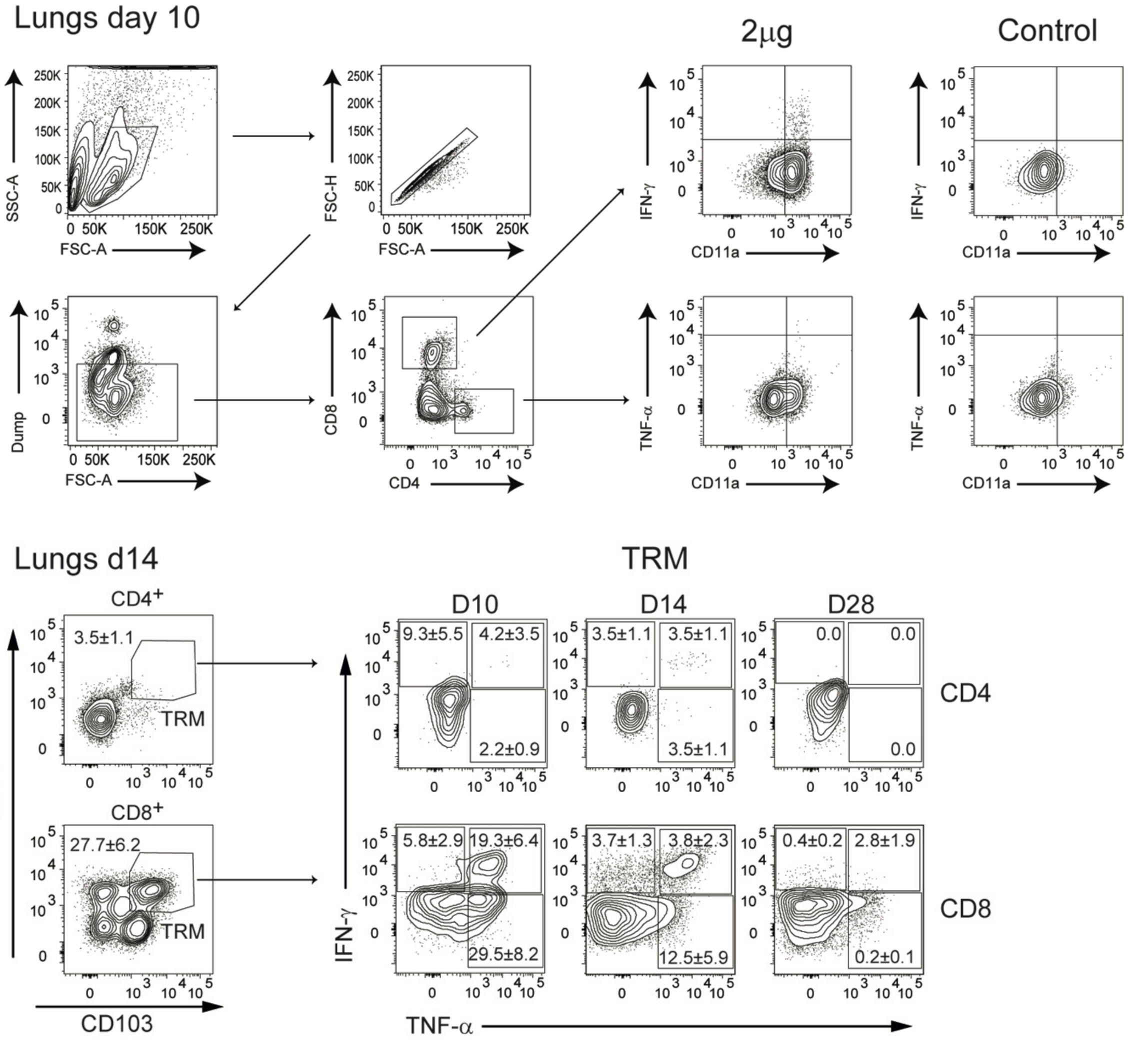

Figure S10. Representative flow cytometry plots for gating of lung Trm cells isolated from mice. Gating strategy to identify cytokine (IFN-γ and TNF-α) producing CD11a^{+} or CD69^{+}CD103^{+} resident memory (Trm) CD4^{+} and CD8^{+} cells.

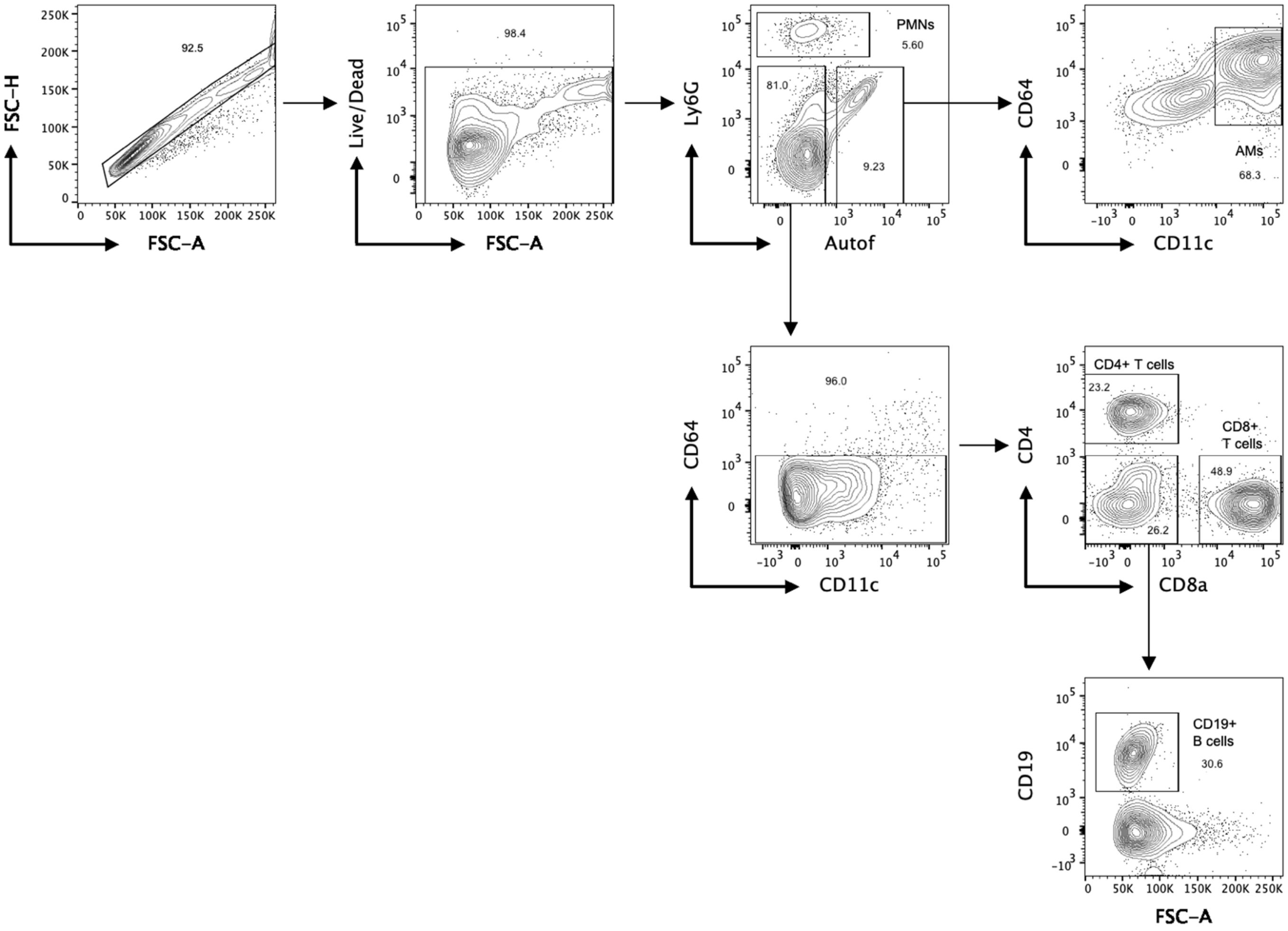

Figure S11. Representative flow cytometry plots for gating of BAL cells isolated from mice. Gating strategy to identify Autof^{+}CD11c^{+}CD64^{+} alveolar macrophages (AMs), CD4^{+} T cells, CD8^{+} T cells or CD19^{+} B cells in the bronchoalveolar (BAL) compartment.
